# An Enhanced Randomized Dung Beetle Optimizer for Global Optimization Problems

**DOI:** 10.3390/biomimetics10110727

**Published:** 2025-11-01

**Authors:** Hui Yu, Mengyuan Xie, Zhanxi Zhou

**Affiliations:** 1 The School of Computer Engineering, Hubei University of Arts and Science, Xiangyang 441053, China; 2 Winchester School of Arts, University of Southampton, Southampton SO23 8DL, UK; 3 Santa Monica College, Santa Monica, CA 90405, USA

**Keywords:** metaheuristic optimization methods, population-based evolutionary strategies, dung beetle optimizer (DBO), combinatorial problem solving, discrete search optimization

## Abstract

The Dung Beetle Optimizer (DBO) has shown promise in solving complex optimization problems, yet it often suffers from premature convergence and limited accuracy. To overcome these limitations, this paper proposes the Enhanced Reproductive Dung Beetle Optimizer (ERDBO). The ERDBO introduces a three-stage mechanism: (1) a larval growth phase using experiential learning to enrich population diversity and improve global exploration; (2) a reproduction and nurturing phase that employs parent–offspring verification and a teaching strategy to strengthen local exploitation; and (3) a predator avoidance phase integrating Lévy flight and sinusoidal perturbations to enhance adaptability and accelerate convergence. The effectiveness of the proposed algorithm is assessed using the CEC2017 benchmark functions, where it is contrasted with several advanced metaheuristic approaches. The experimental findings highlight its advantages in terms of convergence rate, stability, and solution precision. Furthermore, the ERDBO is applied to three well-known engineering design tasks—namely the tension/compression spring, the three-bar truss, and the pressure vessel problem. The outcomes verify both its efficiency and applicability, indicating that the ERDBO provides a robust and competitive optimization framework for tackling challenging real-world engineering scenarios.

## 1. Introduction

Optimization plays a pivotal role in modern science and engineering, serving as the foundation for decision-making, resource allocation, and system design across a broad spectrum of disciplines. Many real-world problems [1,2,3,4], ranging from structural design and mechanical engineering to transportation systems and industrial scheduling, can be formulated as optimization tasks. These problems often involve conflicting objectives, nonlinear constraints, high-dimensional search spaces, and intricate interactions among variables, making their resolution highly challenging. Conventional optimization techniques, such as gradient-based methods or deterministic heuristics, typically suffer from premature convergence or limited applicability in such complex domains, especially when the objective functions are multimodal, discontinuous, or analytically intractable [5,6,7,8]. Consequently, metaheuristic algorithms have emerged as powerful and flexible alternatives, offering robust performance in solving a wide variety of global optimization problems.

Over recent decades, numerous metaheuristic algorithms inspired by natural phenomena, biological processes, and physical laws have been developed. Representative approaches encompass Genetic Algorithms (GAs) [9], Particle Swarm Optimization (PSO) [10], differential evolution (DE) [11], the Artificial Bee Colony (ABC) [12], the Grey Wolf Optimizer (GWO) [13], the Butterfly Optimization Algorithm (BOA) [14], the Sparrow Search Algorithm (SSA) [15], the Chimp Optimization Algorithm (ChOA) [16], the Arithmetic Optimization Algorithm (AOA) [17], the Optical Microscope Algorithm, the Sine Cosine Algorithm [18], and the Artificial Jellyfish Search Optimizer (JS) [19]. These methods rely on stochastic search principles to maintain a balance between global exploration and local exploitation, which helps them to avoid premature convergence and approximate near-optimal solutions. In recent years, newer swarm-intelligence and evolutionary models such as the Whale Optimization Algorithm (WOA) [20], Harris Hawks Optimization (HHO) [21], and the Dung Beetle Optimizer (DBO) [22] have gained considerable interest owing to their conceptual simplicity, adaptability, and promising performance across various benchmark tasks. Nevertheless, many existing algorithms still suffer from drawbacks, including limited convergence efficiency, restricted scalability, and reduced robustness in solving large-scale or constrained optimization problems. Therefore, the pursuit of enhanced metaheuristic frameworks continues to be a vital and evolving research direction.

Among these approaches, the DBO has emerged as a promising algorithm due to its biological inspiration from the unique behaviors of dung beetles, including foraging, breeding, and obstacle avoidance. The algorithm has demonstrated effectiveness in striking a balance between exploration and exploitation. Nevertheless, the canonical DBO still exhibits certain drawbacks, such as susceptibility to local optima and relatively slow convergence in complex search landscapes. To overcome these issues, researchers have explored various extensions and hybridizations of the DBO, integrating adaptive mechanisms, chaos-based initialization, or hybrid operators from other algorithms [4,23,24,25,26]. Nevertheless, significant opportunities for advancement still exist, especially in accelerating convergence, improving accuracy, and strengthening robustness when addressing a variety of engineering optimization tasks.

To address these challenges, this paper proposes the Enhanced Randomized Dung Beetle Optimizer (ERDBO), which introduces two major innovations to refine the balance between global exploration and local exploitation. First, a randomized breeding strategy is incorporated by introducing stochastic factors, modeled as “random spores,” to enhance population diversity and mitigate the risk of premature convergence. Second, sinusoidal perturbation operators, derived from trigonometric functions, are employed to strengthen local exploitation around promising solutions, thereby accelerating convergence while maintaining accuracy. Together, these mechanisms enable the ERDBO to dynamically adapt to varying search landscapes and improve its overall performance in complex optimization scenarios.

The effectiveness of the ERDBO is comprehensively evaluated using the CEC2017 benchmark suite, which consists of a wide range of unimodal, multimodal, hybrid, and composite functions at both 30-dimensional and 100-dimensional scales. Comparative results against several state-of-the-art metaheuristic algorithms demonstrate that the ERDBO consistently achieves superior performance in terms of mean objective value, standard deviation, and robustness. These findings highlight the algorithm’s ability to deliver high-quality solutions with reduced computational effort, particularly in large-scale and high-dimensional optimization problems.

Apart from standard benchmark assessments, the ERDBO’s effectiveness is also demonstrated on a set of widely studied engineering design challenges, including the tension/compression spring, three-bar truss, and pressure vessel problems. These classical engineering problems, characterized by nonlinear constraints and practical design trade-offs, provide a rigorous test bed for assessing algorithmic performance. The experimental results confirm that the ERDBO is capable of producing competitive, and in many cases superior, solutions compared to existing algorithms while maintaining computational efficiency. This underscores the algorithm’s potential for broader use in engineering practice, where reliability, accuracy, and efficiency are crucial.

The contributions of this paper can be summarized as follows:

- Three-Stage Bio-Inspired Mechanism: This study proposes the Enhanced Reproductive Dung Beetle Optimizer (ERDBO), which simulates the complex biological behaviors of dung beetles during the larval growth, reproduction and nurturing stages, and predator avoidance. The mechanism effectively balances global exploration and local exploitation in the search space.
- Innovations in Larval Growth and Reproduction Phases: During the larval growth phase, experiential learning is used to dynamically update individual positions, enhancing global exploration. In the reproduction and nurturing phase, a parent–offspring verification and teaching mechanism is incorporated to guide systematic solution updates, optimize local exploitation, and maintain population integrity.
- Predator Avoidance and Adaptive Displacement: The predator avoidance phase simulates the rapid escape and warning behaviors of dung beetles, enabling adaptive position updates. This strategy enhances population diversity and local search capability, thus improving convergence speed and solution accuracy in complex optimization problems.

The structure of this paper is organized as follows. Section 1 provides a review of the recent developments in metaheuristic algorithms, emphasizing both the advantages and limitations of the DBO algorithm. Section 2 describes the basic procedure of the DBO. In Section 3, the enhanced version, the ERDBO, is introduced along with its key design improvements. Section 4 presents a comparative study of the ERDBO against several classical algorithms via the CEC2017 benchmark suite, including statistical analyses and performance rankings. Section 5 demonstrates the practical utility of the ERDBO by applying it to three representative engineering design problems: the tension/compression spring, the three-bar truss, and the pressure vessel. Finally, Section 6 concludes the paper by summarizing the contributions of the ERDBO and discussing potential future research directions for DBO-based optimization methods.

## 2. Dung Beetle Optimizer

To systematically tackle the problem, the objective function and the set of feasible solutions are initially formulated.

(1) $$\begin{array}{c} \min\{f(x)|x\in X,X\subseteq S\} \end{array}$$

Let f(x) denote the objective function, where x∈X and *X* represents the feasible solution set. The overall search domain is denoted by *S*. Within this framework, *X* is also regarded as the population matrix that includes all candidate solutions, which can be expressed as

(2) $$X={\left[\begin{array}{cccc} x_{1}^{1} & x_{2}^{1} & \ldots & x_{dim}^{1}\\ x_{1}^{2} & x_{2}^{2} & \ldots & x_{dim}^{2}\\ \vdots & \vdots & \ddots & \vdots \\ x_{1}^{N} & x_{2}^{N} & \ldots & x_{dim}^{N} \end{array}\right]}_{N\times \dim}$$

where *N* is the number of individuals in the population, and *d* denotes the dimensionality of the problem space. For experimental validation, the proposed method is tested on the CEC2017 benchmark functions with N=30, while the problem dimension is set to d=30 or d=100 in order to examine its scalability across different search spaces. The initial population is generated randomly, with each individual corresponding to a point in the feasible domain, thus serving as a candidate solution. Accordingly, a solution vector *x* can be represented as

(3) $$\begin{array}{c} x_{j}^{n}=lb+\mathrm{rand}\times \left(ub-lb\right),n=1,\;2,\;\ldots ,\;N\;\mathrm{and}\;j=1,\;2,\;\ldots ,\;\dim \end{array}$$

For the optimization variables, the lower and upper limits, represented as lb and ub, are specified by the CEC2017 benchmark to lie within [−100,100]; i.e., lb=−100 and ub=100. To initialize the population, the function rand is employed to produce random values uniformly distributed over [0,1], thereby introducing a stochastic element into the initialization procedure.

### 2.1. Rollerball Dung Beetle

In their natural environment, dung beetles demonstrate an impressive capability to traverse linear trajectories while rolling dung balls, frequently exposed to strong sunlight. To capture this sophisticated navigational behavior, the position update of the beetle is described by Equation (4), following the original formulation. This expression explicitly dictates how the beetle’s position is adjusted throughout the rolling process.

(4) $$\begin{array}{cc} \; & x_{i}\left(t+1\right)=x_{i}\left(t\right)+a\cdot k\cdot x_{i}\left(t-1\right)+b\cdot ∆x\\ \; & ∆x=\left|x_{i}\left(t\right)-X^{worst}\right| \end{array}$$

Within the proposed framework, the variable *t* corresponds to the current iteration index, and $x_{i}\left(t\right)$ denotes the position of the *i*th dung beetle at iteration *t*. The parameter *a* governs whether the beetle follows its original trajectory or deviates from it. Specifically, *a* is assigned a binary value: a=1 indicates adherence to the original path, whereas a=−1 represents a deviation from it.

The parameter k∈(0,0.2] acts as a defect coefficient, initially set to 0.1, controlling the magnitude of deviation. The constant b∈[0,1], chosen as 0.3 in this study, modulates the influence of random environmental perturbations. Both *a* and *k* are treated as fixed parameters, and their product with $x_{i}\left(t\right)$ contributes directly to updating the agent’s position.

The term $X_{\mathrm{worst}}$ represents the location of the worst-performing individual in the population, while ∆*x* captures the effect of solar illumination. Larger values of ∆*x* correspond to increased separation between the dung beetle and the light source.

In natural habitats, dung beetles exhibit adaptive responses to obstacles by performing characteristic turning maneuvers, often described as dance-like behaviors, to reorient their rolling paths. To mimic this phenomenon, the original study introduced a probabilistic mechanism to approximate the likelihood of obstacle encounters during the rolling process. Upon detecting an obstacle, the beetle’s updated direction is determined via a tangent-based function, reproducing the biologically inspired adjustment in trajectory.

This adaptive behavior is incorporated into the position update described by Equation (5), which governs the motion dynamics of the rolling dung beetle. Integrating this mechanism reflects the inherent flexibility in the beetle’s navigation and provides a biologically grounded justification for the employed position update strategy.

(5) $$x_{i}\left(t+1\right)=x_{i}\left(t\right)+\tan\left(\theta \right)\left|x_{t}\left(t\right)-x_{i}\left(t-1\right)\right|$$

**Figure 1:**
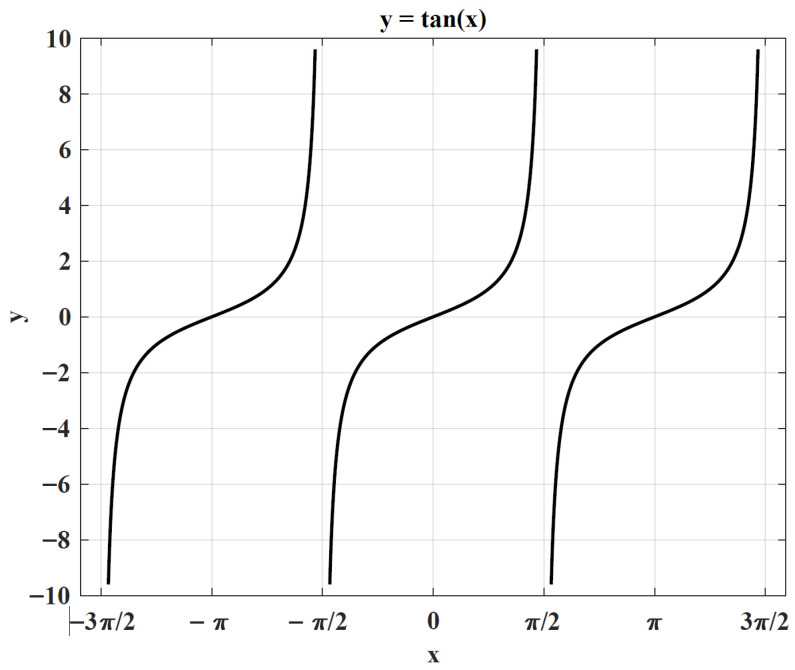
The value of tan(x).

The deflection angle θ is confined to the range [0,π]. Position updates are skipped when θ takes the values 0, $\frac{\pi }{2}$, or π. It is important to note that, as θ approaches $\frac{\pi }{2}$ from the left ($\theta \to {\frac{\pi }{2}}^{-}$), tanθ tends to positive infinity, whereas approaching from the right ($\theta \to {\frac{\pi }{2}}^{+}$) results in tanθ tending to negative infinity. This unbounded behavior of tanθ may cause the computation of $x_{i}\left(t\right)$ to become unstable or undefined. Moreover, at θ=π, since tanθ=0, the position $x_{i}\left(t+1\right)$ remains unchanged. The dependency of tan(θ) on θ is depicted in Figure 1.

### 2.2. Spawning Dung Beetles

In their natural environment, dung beetles select a secure site for oviposition. To mimic this behavior, the original DBO algorithm employs a boundary selection mechanism to define the oviposition region, as shown in Equation (6):

(6) $$\begin{array}{cc} \; & Lb^{*}=\max\left(X_{\mathrm{best}1}\times \left(1-R\right),Lb\right)\\ \; & Ub^{*}=\min\left(X_{\mathrm{best}1}\times \left(1-R\right),Ub\right) \end{array}$$

Here, $Lb^{*}$ and $Ub^{*}$ denote the lower and upper bounds of the oviposition region, respectively. $X_{\mathrm{best}1}$ represents the position of the top-performing individual in the population, and the coefficient *R* is defined as $R=1-\frac{t}{T_{\max}}$, with $T_{\max}$ being the maximum number of iterations.

Once the optimal oviposition region is established, dung beetles lay their eggs within this area. Each egg-laying event updates the agent’s position, enabling local exploration around the current best solution and reducing the risk of premature convergence. The position update formula for spawning dung beetles is provided in Equation (7):

(7) $$x_{i}\left(t+1\right)=X_{\mathrm{best}1}+b_{1}⊙\left(x_{i}\left(t\right)-Lb^{*}\right)+b_{2}⊙\left(x_{i}\left(t\right)-Ub^{*}\right)$$

In this work, $b_{1}$ and $b_{2}$ are stochastic vectors of size 1×Dim, where Dim denotes the dimensionality of the optimization problem. The Hadamard product (⊙) is applied element-wise between $b_{1}$ and $\left(x_{i}\left(t\right)-Lb^{*}\right)$, as well as between $b_{2}$ and $\left(x_{i}\left(t\right)-Ub^{*}\right)$.

### 2.3. Foraging Dung Beetles

In their natural habitat, foraging dung beetles exhibit behavior analogous to selecting secure oviposition sites. The designated region is defined in the original study by the following formulation, as presented in Equation (8):

(8) $$\begin{array}{cc} \; & Lb^{b}=\max\left(X^{best2}\times \left(1-R\right),Lb\right)\\ \; & Ub^{b}=\min\left(X^{best2}\times \left(1-R\right),Ub\right) \end{array}$$

In this context, $X^{best2}$ represents the globally optimal position, while $Lb^{b}$ and $Ub^{b}$ specify the lower and upper boundaries of the optimal foraging region, respectively. Additionally, Lb and Ub denote the overall lower and upper bounds of the problem domain. Each foraging operation carried out by the dung beetle induces a position update, which is described by Equation (9):

(9) $$x_{i}\left(t+1\right)=x_{i}\left(t\right)+C_{1}\times \left(x_{i}\left(t\right)-Lb^{b}\right)+C_{2}\times \left(x_{i}\left(t\right)-Ub^{b}\right)$$

The variable $C_{1}$ is sampled from a normal distribution, whereas $C_{2}$ is a 1×Dim vector with elements independently drawn from a uniform distribution over [0,1]. The vector $C_{1}$ is applied element-wise (Hadamard product) to the difference $\left(x_{i}\left(t\right)-Lb^{*}\right)$, while $C_{2}$ is similarly multiplied element-wise with $\left(x_{i}\left(t\right)-Ub^{*}\right)$.

### 2.4. Stealing Dung Beetles

In natural environments, some dung beetles exhibit the behavior of stealing dung balls from conspecifics. To capture this phenomenon, the original study defines the global best position, denoted by *X*, to represent the location of the contested dung ball. The corresponding stealing behavior is then modeled as a position update, which is mathematically formulated by the following equation:

(10) $$x_{i}\left(t+1\right)=X^{best2}+S\cdot g\cdot \left(\left|x_{i}\left(t\right)-X^{best1}\right|+\left|x_{i}\left(t\right)-X^{best2}\right|\right)$$

According to the original study, the parameter *S* is a constant with a value of 0.5. The variable *g* represents the magnitude of a random vector, and Dim denotes the dimensionality of the optimization problem. In this context, *S* is treated as a scalar, *g* as a column vector, and $x_{i}\left(t\right)$ as a vector; their dot product yields a resultant vector.

Furthermore, the study specifies the population sizes for the different categories of dung beetles as follows: 6 rollers, 6 breeders, 7 foragers, and 11 thieves.

### 2.5. Pseudocode for the DBO Algorithm

The pseudocode for DBO is as follows:

### 2.6. The Time Complexity of DBO

At the beginning, the population is initialized and the algorithm parameters are set, resulting in a computational cost of O(N·D), where *N* denotes the population size and *D* indicates the dimensionality of the problem. In each iteration, updating the positions of all dung beetles—including rollers, spawners, foragers, and thieves—also requires O(N·D) operations. Considering that the algorithm runs for $T_{\max}$ iterations, the overall time complexity amounts to $O(T_{\max}\cdot N\cdot D)$.

## 3. Enhanced Reproductive Dung Beetle Optimizer

### 3.1. Our Motivation

This study introduces the ERDBO Algorithm 1, inspired by the complex biological processes observed in dung beetles during their growth, reproduction, and predator avoidance phases.

Firstly, in the larval growth stage, population members’ positions are dynamically updated through a process of experiential learning. Considering the large population size and survival challenges, the algorithm simulates rapid larval development by continuously assimilating experiential knowledge to adjust individual positions within the solution space. This mechanism models a sequence of motion patterns that induce significant positional diversity, thereby enhancing exploration capabilities.
**Algorithm 1** Framework of the Dung Beetle Optimizer (DBO) Algorithm**Input:** Maximum number of iterations $T_{\max}$, population size *N*
**Output:** Optimal solution $X^{best2}$ and its corresponding fitness value $f_{\min}$
  1:Initialize a population of *N* individuals and set the necessary parameters.  2:**while**$\;t\leq T_{\max}\;$**do**  3:    **for** each individual in the rolling dung beetle group **do**  4:        Generate a random number a=rand(1)  5:        **if** a≤0.9 **then**  6:            Update the position of the rolling beetle according to Equation (4)  7:        **else**  8:            Perform position update simulating obstacle-influenced rolling using Equation (5)  9:        **end if**10:    **end for**11:    Compute the nonlinear convergence factor $R=1-t/T_{\max}$12:    **for** each individual in the spawning dung beetle group **do**13:        Update the beetle’s position using Equations (6) and  (7)14:    **end for**15:    **for** each individual in the foraging dung beetle group **do**16:        Update the beetle’s position according to Equations (8) and  (9)17:    **end for**18:    **for** each individual in the stealing dung beetle group **do**19:        Update the beetle’s position based on Equation (10)20:    **end for**21:**end while**22:**return** Optimal position $X^{best2}$ and its associated fitness $f_{\min}$


Secondly, during the reproduction and nurturing stage, the ERDBO incorporates a biologically inspired teaching mechanism. When the environmental risk threshold is low, dung beetles enter the breeding phase, wherein a unique parentage verification process during oviposition prevents external species infiltration. This mechanism ensures population integrity and guides the systematic update of solution candidates’ positions.

Thirdly, in the foraging phase, the algorithm simulates dung beetles’ predator avoidance behaviors to adaptively update positions. Upon detection by predators, the targeted beetle executes rapid escape maneuvers accompanied by disruptive leg movements and emits warning signals to alert conspecifics. Consequently, the targeted individual undergoes minor positional adjustments, while other members perform evasive hovering, resulting in substantial spatial displacement. This adaptive strategy improves the algorithm’s ability to avoid premature convergence and maintain population diversity.

Together, these biologically inspired strategies establish a robust balance between exploration and exploitation, contributing to improved convergence speed and solution accuracy.

### 3.2. The Larval Growth Stage of Dung Beetles

During the larval growth phase, the positions of individuals in the population are adaptively adjusted based on experiential learning strategies [27,28,29]. Considering the large population size and competitive survival pressures, the algorithm simulates the rapid development of larvae by continuously incorporating accumulated experiential knowledge to dynamically update each individual’s position within the search space. This mechanism effectively models a series of complex movement behaviors, thereby increasing positional diversity and enhancing the exploratory capability of the algorithm. The position update during the larval growth stage is expressed by Equation (11):

(11) $$x_{i}\left(t+1\right)=x_{i}\left(t\right)+\left(Lb^{b}+\left(Ub^{b}-Lb^{b}\right)\times \mathrm{rand}\right),\;r>0.5$$

### 3.3. The Reproduction and Nurturing Stage

In the second phase of dung beetle reproduction, the positions of population members are updated by simulating the teaching mechanisms observed during the beetles’ reproductive and nurturing behaviors. When the environmental risk threshold is low, dung beetles enter the breeding stage and employ a distinctive parent–offspring identification strategy during oviposition to prevent invasion by foreign species.

(12) $$\begin{array}{cc} \; & s=r_{1}*20+r_{2}*20\\ \; & x_{i}\left(t+1\right)=X_{G}+\left(X^{best2}*x_{i}\left(t\right)\right)\times p,\;r<0.5\;\mathrm{and}\;s<20\\ \; & X_{G}=X_{b}*0.8\\ \; & p=\sin\left(Ub^{b}-Lb^{b}\right)*2+\left(Ub^{b}-Lb^{b}\right)*m\\ \; & m=\left(\frac{j}{N}\right)*2 \end{array}$$

Here, $r_{1}$ and $r_{2}$ are random variables drawn from a normal distribution.

During the predator avoidance phase, the positions of population members are updated based on the defensive mechanisms that dung beetles employ to resist predator attacks. When an exceptional dung beetle is spotted by a predator, it responds by rapidly fleeing and repeatedly striking its legs to obscure the predator’s vision. Simultaneously, it emits warning signals to alert other members of the population.

Under such circumstances, the targeted dung beetle escapes quickly, resulting in a slight positional change, while the remaining members perform evasive maneuvers to avoid the predator, leading to significant positional shifts. These two distinct behavioral responses within the DBO population enhance the algorithm’s ability to expand its search range in the solution space while improving its local exploitation capabilities. The corresponding motion patterns are mathematically defined in Equation (13):

(13) $$\begin{array}{cc} \; & x_{i}\left(t+1\right)=X^{best2}\times l\times k,\;r<0.5\;\;\mathrm{and}\;\;s>20\\ \; & k=0.2\times \sin\left(\frac{\pi }{2}-w\right)\\ \; & w=\frac{\pi }{2}\times \frac{j}{N} \end{array}$$

Here, *l* denotes the Lévy flight-based random step size, which enables the algorithm to escape from local optima. The parameter *k* represents the adaptive flight balance coefficient, while *w* denotes the calling frequency value.

### 3.4. Implementation of ERDBO

Population Initialization: The algorithm starts by setting the population size *N*, the problem dimensionality *D*, the maximum number of evaluations Max, and the upper and lower bounds ub and lb. The initial population *x* is then generated within the search space following Equation (3).

Larval Growth Stage: During the growth phase, dung beetles continuously adjust their positions to assimilate extensive experiential knowledge, thereby facilitating rapid development. To simulate this, a foundational growth model is constructed, enabling dung beetles to perform wide-ranging exploration within a constrained solution space. Position updates in this phase are computed using Equation (11).

Reproduction and Nurturing Stage: When environmental conditions are deemed safe, dung beetles engage in educational behaviors during reproduction, wherein several members take turns caring for the eggs until hatching. This cooperative mechanism results in localized movements near the nesting area as each member alternates between foraging and caregiving. Based on this, a localized exploitation model is established, allowing dung beetles to perform in-depth exploration in defined regions. The corresponding position update is described by Equation (12).

Predator Avoidance Stage: In response to perceived threats, dung beetles shake their legs to distract predators and issue warning signals to nearby members. This defensive behavior introduces greater randomness into their exploration patterns. A predator avoidance model is established to simulate this adaptive strategy, and the associated position update is formalized in Equation (13).

(14) $$\begin{array}{c} x_{i}\left(t+1\right)=\left\{\begin{array}{c} x_{i}\left(t\right)+\left(Lb^{b}+\left(Ub^{b}-Lb^{b}\right)\times rand\right),\;r>0.5\\ X_{G}+\left(X^{best2}*x_{i}\left(t\right)\right)\times p,\;r<0.5\;\mathrm{and}\;s<20\\ X^{best2}\times l\times k,\;r<0.5\;\;\mathrm{and}\;\;s>20 \end{array}\right. \end{array}$$

### 3.5. Pseudocode for the ERDBO Algorithm

The pseudocode for the ERDBO algorithm is in Algorithm 2. The value of *a* is a uniformly distributed random real number greater than or equal to 0 and less than 1.
**Algorithm 2** Framework of the ERDBO Algorithm**Input:** Maximum number of iterations $T_{\max}$, population size *N*
**Output:** Optimal solution $X^{best2}$ and its associated fitness value $f_{\min}$
  1:Initialize a population of *N* individuals and configure algorithm parameters.  2:**while**$\;t\leq T_{\max}\;$**do**  3:    **for** each individual in the rolling dung beetle group **do**  4:        Generate a random scalar a=rand(1)  5:        **if** a≤0.9 **then**  6:            Update the position according to Equation (4)  7:        **else**  8:            Perform obstacle-influenced rolling update using Equation (5)  9:        **end if**10:    **end for**11:    Compute the nonlinear convergence factor $R=1-t/T_{\max}$12:    **for** each individual in the spawning dung beetle group **do**13:        Update the position using Equations (6) and  (7)14:    **end for**15:    **for** each individual in the foraging dung beetle group **do**16:        Update the position according to Equation (14)17:    **end for**18:    **for** each individual in the stealing dung beetle group **do**19:        Set reproductive or interaction parameters20:        Update the position based on Equations (12) and (13)21:    **end for**22:**end while**23:**return** Optimal position $X^{best2}$ and its corresponding fitness $f_{\min}$


### 3.6. The Time Complexity of ERDBO

The computational complexity of the ERDBO algorithm is primarily determined by the population size and the total number of iterations. Let $T_{\max}$ denote the maximum number of iterations, *N* the population size, and *D* the dimensionality of each candidate solution.

During each iteration, individuals are categorized into four behavioral types: rollers, spawners, foragers, and thieves. Each individual updates its position according to specific rules, with each update requiring constant-time operations (i.e., O(1)), possibly involving vector operations across *D* dimensions.

Consequently, the cost per iteration scales with both the population size and the solution dimensionality. Considering that the algorithm executes for $T_{\max}$ iterations, the total time complexity of the ERDBO can be expressed as $O(T_{\max}\cdot N\cdot D)$.

This analysis shows that the algorithm exhibits linear scalability with respect to the number of iterations, the population size, and the problem dimensionality.

## 4. Experimental Results and Discussion

In this study, the performance of the proposed enhanced algorithm is systematically evaluated using the CEC2017 benchmark suite [30,31]. A comprehensive overview of the benchmark functions is provided in Table 1. Since the original F2 function is excluded from the CEC2017 set, a total of 29 single-objective benchmark functions are employed for the evaluation.

Specifically, F1 and F2 are unimodal functions, each featuring a single global optimum. Functions F3 through F9 are simple multimodal functions containing multiple local optima. Functions F10 to F19 are hybrid functions, each composed of three or more rotated or shifted CEC2017 benchmark functions. Finally, functions F20 to F29 are composite functions formed by combining at least three hybrid or transformed benchmark components, incorporating both rotation and translation operations.

The comparative algorithms selected for performance evaluation include SSA, HHO, BOA, OMA (only for the CEC2017 benchmark functions, as clarified in Dr. Moh Nur Sholeh’s work), SCA, and DBO. To ensure fair comparison, the initial population size for all the algorithms is uniformly set to 30, with a maximum of 500 iterations. To reduce the influence of randomness, each algorithm is executed independently for 30 runs, and the results are subjected to statistical analysis. The parameter settings for all the algorithms are detailed in Table 2.

### 4.1. CEC2017 Test Function Results and Analysis

The statistical outcomes for the CEC2017 benchmark functions in 30- and 100-dimensional settings are systematically recorded. The metrics reported include the minimum (min), mean, and standard deviation (std) calculated over 100 independent runs for each algorithm. The best mean value for each test function is highlighted in bold. The final row, labeled “Total,” summarizes the number of instances in which each algorithm achieved the highest mean performance across all the benchmark functions. Detailed results for the 30- and 100-dimensional cases are presented in Table 3 and Table 4, respectively.

An analysis of Table 3 and Table 4 reveals that, in the 30-dimensional experiments, the ERDBO achieved the top-ranked solution in 14 of the 29 benchmark functions, reflecting its overall effectiveness. For the other 15 functions, although the absolute optimum was not reached, the ERDBO consistently provided high-quality suboptimal solutions, demonstrating its capability to handle a diverse set of problems. In the 100-dimensional experiments, a similar pattern is observed: the ERDBO secured the best result in 14 functions and produced robust suboptimal outcomes for the remaining cases. These findings indicate that the ERDBO maintains stable performance and robustness even in high-dimensional optimization scenarios. The subsequent sections present a more detailed discussion of these results.

- For the unimodal function F1, the ERDBO showed competitive results but was slightly outperformed by WOA in both 30- and 100-dimensional settings and therefore did not achieve the top rank among all the tested algorithms. In contrast, for the unimodal function F2, the ERDBO consistently attained the fifth position across both dimensions, closely following SCA and WOA while clearly outperforming the original DBO. These observations suggest that the ERDBO provides a substantial enhancement over the baseline DBO, demonstrating stable and reliable convergence behavior on unimodal optimization tasks.
- For the simple multimodal functions F3–F9, the ERDBO demonstrated strong performance in the 30-dimensional experiments, achieving the highest rank in F3, F4, F6, F7, and F9. These results reflect the algorithm’s improved exploration ability and its effectiveness in avoiding local optima. Although the ERDBO was slightly outperformed by WOA and SCA in F5 and F8, it still significantly exceeded the original DBO and most other competing algorithms. In the 100-dimensional experiments, the ERDBO continued to perform well, attaining the top position in F4, F5, F8, and F9, which illustrates its robustness and scalability in higher-dimensional search spaces. While WOA and SCA occasionally achieved better results on specific functions, the ERDBO consistently produced competitive outcomes across the majority of the benchmarks. The overall advantage of the ERDBO over DBO further confirms the efficacy of the proposed algorithmic improvements.
- Regarding the hybrid functions F10–F19, the ERDBO achieved the highest ranks in F11, F15, and F17 under the 30-dimensional setting, showcasing its capability in handling complex problem landscapes. Although it slightly trailed WOA on several functions, the ERDBO outperformed the basic DBO by a substantial margin, highlighting the effectiveness of its hybrid optimization strategies. In the 100-dimensional scenario, the ERDBO continued to perform competitively, ranking prominently in F11, F15, F16, and F19 despite minor performance gaps relative to WOA and SCA. These findings demonstrate the ERDBO’s adaptability and improved solution quality in multidimensional hybrid problems.
- For the composite functions F20–F29, the ERDBO achieved notable performance in the 30-dimensional experiments, securing the top rank in F20, F22–F24, and F26. This demonstrates its capability in handling complex and multi-component optimization landscapes. While WOA slightly outperformed the ERDBO on a few functions, the ERDBO still markedly surpassed the original DBO, highlighting its enhanced efficiency and accuracy in optimizing composite functions. In the 100-dimensional experiments, the ERDBO continued to exhibit strong results, attaining the highest rank in F20–F23, F26, and F28, further illustrating its robustness and scalability in high-dimensional search spaces. Overall, these findings confirm that the ERDBO offers significant improvements in convergence speed, solution quality, and algorithmic stability across a diverse set of benchmark problems.

The ERDBO demonstrates superior optimization performance across a wide range of benchmark functions owing to multiple key algorithmic enhancements. It introduces an adaptive weight adjustment mechanism that dynamically balances global exploration and local exploitation, substantially improving its ability to escape local optima and enhancing search diversity and efficiency. Additionally, the ERDBO employs an improved heuristic update strategy that optimizes information sharing and maintains population diversity, effectively preventing premature convergence while accelerating convergence speed and improving solution quality. These features ensure robust stability and reliability even in high-dimensional complex optimization problems. Moreover, the ERDBO incorporates automated and intelligent parameter tuning, reducing dependence on user expertise and broadening its applicability across diverse problem domains. Extensive testing on multi-dimensional and varied problem sets confirms its scalability and strong generalization capabilities, from low- to high-dimensional and from simple to complex tasks, providing a solid foundation for real-world engineering and scientific applications. Overall, the ERDBO not only significantly outperforms the original DBO and other benchmark algorithms in convergence speed, accuracy, and stability but also achieves critical breakthroughs in algorithm design, establishing itself as a powerful tool for solving complex, nonlinear, multimodal, and high-dimensional optimization problems and advancing the field of heuristic optimization.

### 4.2. CEC2017 Convergence Curve Analysis

**Figure 2:**
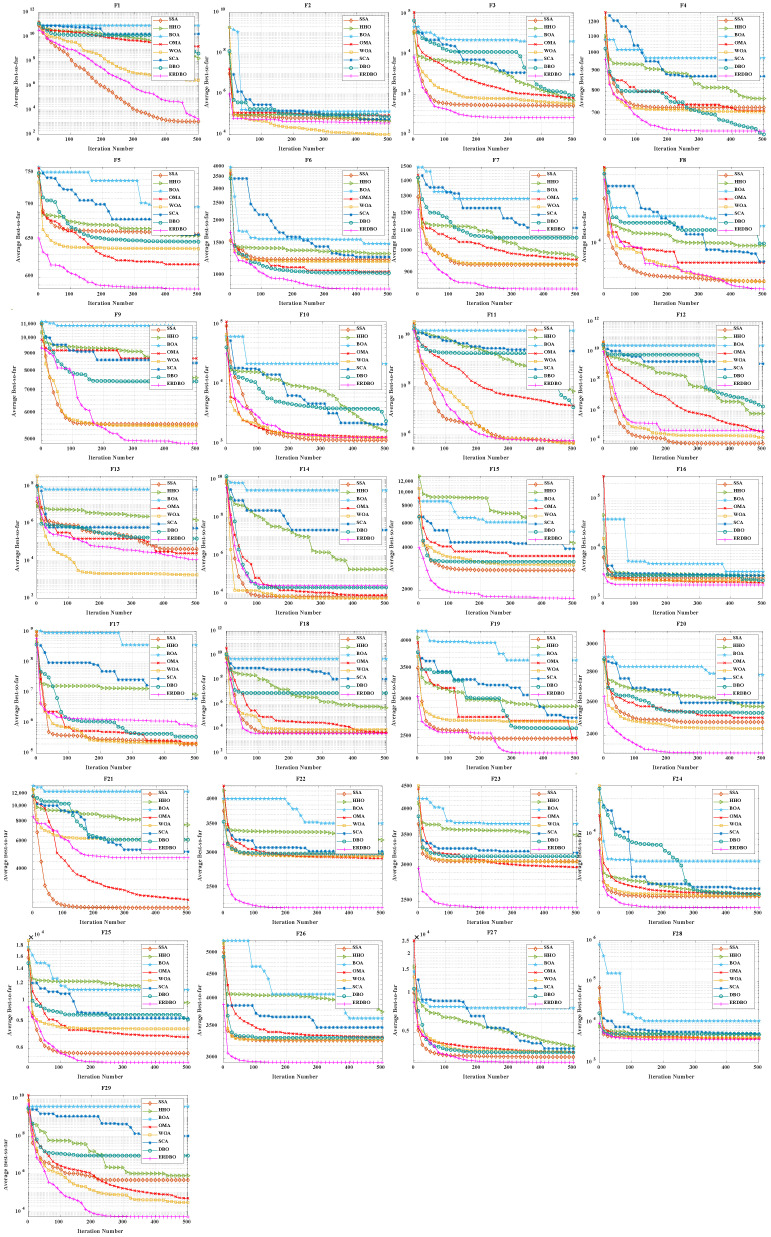
CEC2017 test curve chart (Dim = 30).

**Figure 3:**
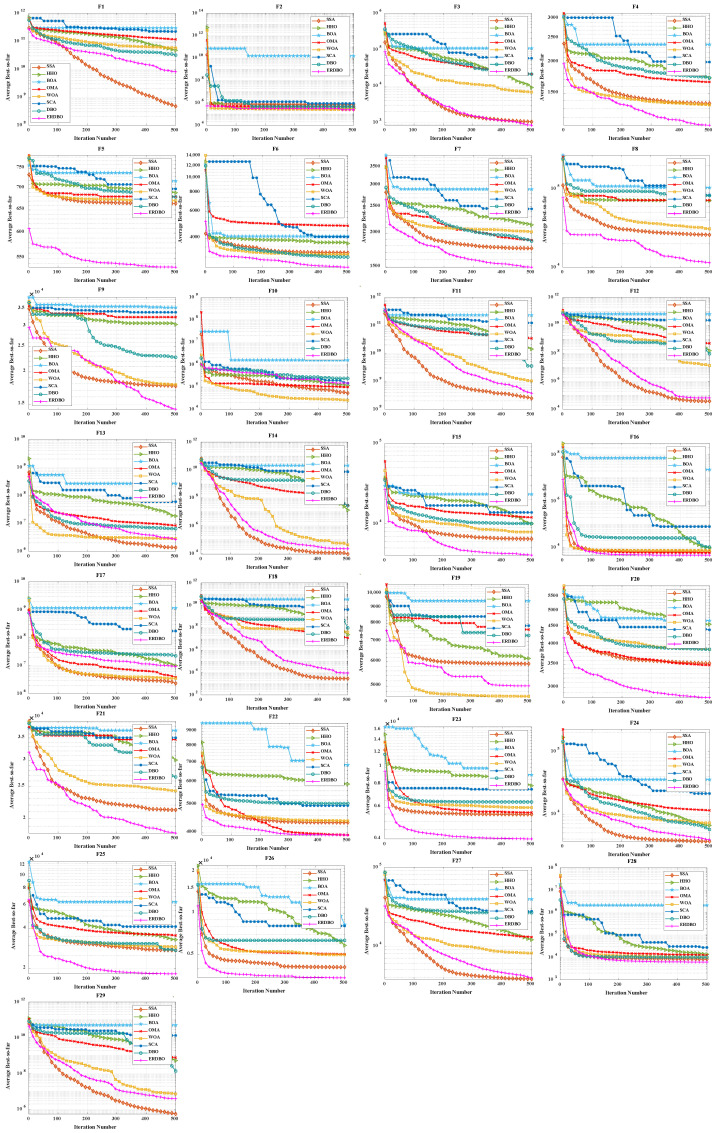
CEC2017 test curve chart (Dim = 100).

To evaluate both convergence speed and solution accuracy, convergence curves were plotted for the 30- and 100-dimensional experiments, comparing the ERDBO with several competing algorithms, as illustrated in Figure 2 and Figure 3. In each subplot, the x-axis represents the iteration count, while the y-axis shows the average objective values obtained over 30 and 100 independent runs. The analysis of these curves yields the following insights:

- For the unimodal function F1, the ERDBO initially converged more slowly than SCA. However, after roughly two-thirds of the iterations, its convergence accelerated, eventually catching up with SCA and surpassing all the other algorithms. This pattern was observed in both 30- and 100-dimensional settings, illustrating the ERDBO’s effective balance between exploration and exploitation throughout the search process. On F2, the ERDBO consistently converged faster than the original DBO, achieving superior final solutions. These observations confirm the ERDBO’s ability to enhance convergence behavior, particularly in the later stages of unimodal optimization.
- For simple multimodal functions F3–F9, the ERDBO achieved rapid convergence and top-ranked solutions in the 30-dimensional case, particularly for F3, F4, F6, F7, and F9. Notably, on F6, the ERDBO reached the equilibrium point of the baseline DBO in approximately 280 iterations, highlighting the efficiency of its improvements. In the 100-dimensional experiments, the ERDBO maintained robust convergence, leading on F4, F5, F8, and F9 while consistently obtaining high-quality solutions. These results demonstrate the ERDBO’s scalability and reliability in high-dimensional multimodal optimization tasks.
- For hybrid functions F10–F19, the ERDBO exhibited consistently fast convergence, frequently leading on F15 and F19. In 30 dimensions, the fastest optimal solutions were observed on F11, F15, F16, and F17. Specifically, the ERDBO achieved equilibrium on F15 and F16 well before 100 iterations, outperforming the other algorithms in both speed and solution quality. In 100-dimensional scenarios, the ERDBO continued to excel, attaining optimal solutions on F15, F16, F17, and F19 with significantly faster convergence than the baseline DBO. These findings highlight the ERDBO’s effectiveness in efficiently handling complex landscapes composed of multiple functional components.
- For composite functions F20–F29, the ERDBO consistently delivered excellent performance in 30 dimensions, rapidly converging to optimal solutions on F20, F22, F23, and F26–F29, outperforming most competing algorithms. Its performance was comparable to SCA only on F28 and F29, emphasizing its competitiveness. In 100-dimensional experiments, the ERDBO maintained its advantage, achieving optimal solutions on F20–F23, F25, and F26 with impressive convergence speed. Overall, these results confirm the ERDBO’s strong adaptability and efficiency in complex high-dimensional composite optimization problems, demonstrating its potential for real-world applications requiring robust optimization.

### 4.3. Wilcoxon Rank-Sum Test

The Wilcoxon rank-sum test, a widely used nonparametric statistical approach [32,33], was applied to assess the performance differences between the proposed ERDBO and conventional algorithms without assuming specific data distributions. In this work, the test was employed to evaluate the performance variations between the ERDBO and six benchmark algorithms. Experiments conducted on the CEC2017 dataset across multiple dimensions provided insights into the relative effectiveness of these algorithms in solving optimization problems.

For the 30-dimensional results (Table 5), the ERDBO exhibited clear advantages over WOA and SAO, likely due to its improved exploration capability. When compared with the DBO and HHO, significant differences were observed across all the test functions, emphasizing the ERDBO’s stability and robustness. Comparisons with WOA and SCA demonstrated the ERDBO’s superiority on several specific functions, although some performance fluctuations were present.

In the 100-dimensional experiments (Table 6), the ERDBO continued to outperform DBO, WOA, and SAO significantly, highlighting its adaptability to high-dimensional problems. However, for SSA, HHO, and other algorithms, differences were observed on certain functions, which can be attributed to variations in search strategies and convergence behaviors in high-dimensional search spaces.

### 4.4. Comparison of ERDBO with Other Improved DBO Variants, DE, and State-of-the-Art Algorithms

**Figure 4:**
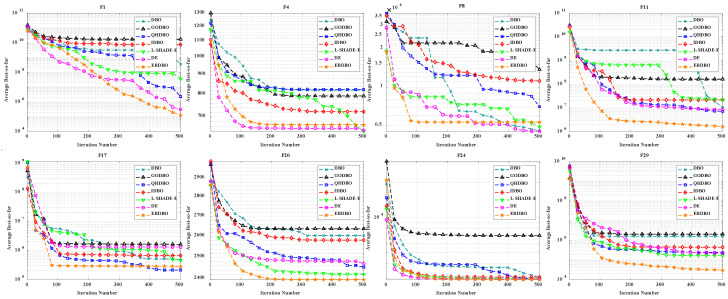
Convergence curves of ERDBO versus other algorithms (Dim = 30).

**Figure 5:**
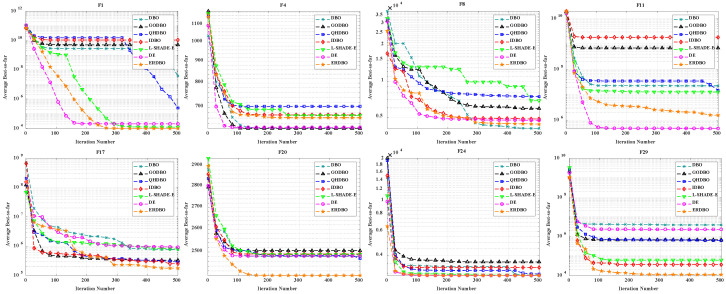
Convergence curves of ERDBO versus other algorithms (Dim = 100).

To thoroughly evaluate the performance of the ERDBO, we conducted extensive experiments, comparing it with the original DBO, several of its improved variants on the CEC2017 benchmark functions with multiple dimensionalities. L-SHADE-E is recognized as one of the state-of-the-art differential evolution variants and is widely used as a performance benchmark in the evolutionary computation community. These benchmark functions cover a wide range of characteristics, including unimodal, multimodal, hybrid, and composition functions, providing a comprehensive test of algorithmic exploration and exploitation capabilities. The convergence behavior of all the algorithms is illustrated in Figure 4 (Dim = 30) and Figure 5 (Dim = 100), which demonstrate that the ERDBO consistently achieves faster convergence and higher-quality solutions across most functions. These results confirm the effectiveness of the biologically inspired strategies incorporated into the ERDBO, particularly in balancing global exploration and local exploitation, and highlight its superiority over both conventional DBO variants and other competitive metaheuristic algorithms.

As shown in Figure 4 and Figure 5, the ERDBO demonstrates superior performance compared to the original DBO, its improved variants, the L-SHADE-E algorithm, and the DE algorithm on several benchmark functions (F1, F4, F8, F11, F20, and F29) of the CEC2017 suite for both Dim = 30 and Dim = 100. In the 30-dimensional case, the ERDBO achieves faster convergence and higher-quality solutions due to its enhanced exploration and exploitation mechanisms, effectively avoiding premature convergence. In the 100-dimensional case, despite the increased search space and higher problem complexity, the ERDBO maintains its advantage by efficiently balancing global exploration and local exploitation, outperforming competing algorithms, such as L-SHADE-E and DE, which often converge slowly or become trapped in local optima. These results collectively highlight the robustness, scalability, and practical effectiveness of the biologically inspired strategies incorporated into the ERDBO across different problem dimensions.

## 5. Engineering Optimization Issues

### 5.1. Tension/Compression Spring-Design Problem

The design optimization of tension/compression springs is a foundational yet complex topic in mechanical engineering [34,35] given the widespread use of springs as indispensable components in a multitude of mechanical and structural systems. Springs play a pivotal role in absorbing energy, providing force, and enabling motion control in systems ranging from automotive suspensions to aerospace actuators. At the heart of this optimization task lies the fine-tuning of critical geometric parameters—including wire diameter, mean coil diameter, and the number of active coils—to achieve a balance between mechanical performance and structural efficiency.

The primary objective in spring design is typically to minimize the spring’s weight while ensuring that it can withstand specified loads and deformations under various working conditions. A reduction in weight not only contributes to the overall efficiency and responsiveness of the mechanical system but also supports the broader goals of lightweight engineering and sustainability. Achieving this requires solving a constrained nonlinear optimization problem in which material strength, allowable stress, and manufacturing constraints must all be carefully considered.

In practice, engineers must integrate multiple design considerations, including the mechanical behavior of spring materials, fatigue resistance, space limitations, and thermal or environmental factors. The trade-offs between these competing requirements demand a systematic and intelligent optimization approach, one that can navigate the complex design landscape to identify globally optimal solutions. Consequently, the tension/compression spring-design problem serves as a benchmark for testing both algorithmic performance and engineering insight.

Addressing this problem effectively has significant implications across industries. Optimized spring designs lead to lighter, more compact, and cost-effective components, directly contributing to improved energy efficiency, lower material usage, and enhanced system durability. Such advancements are especially valuable in high-performance sectors like aerospace and automotive engineering, where weight reduction is closely linked to fuel efficiency and system responsiveness. Additionally, efficient spring designs can reduce manufacturing complexity and improve product reliability, thereby supporting sustainable and economical production practices.

**Figure 6:**
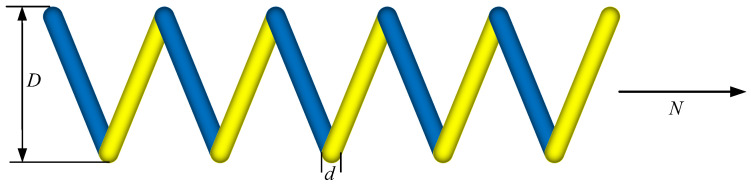
Extension (compression) spring-design issues.

Figure 6 illustrates the schematic of the tension/compression spring optimization problem, where the design variables—wire diameter (*d*), mean coil diameter (*D*), and number of active coils (*N*)—are adjusted to minimize the spring’s total mass. The goal is to ensure that the resulting spring meets all the performance requirements under operational loads while achieving the lightest possible configuration. This kind of optimization framework not only yields high-performance and lightweight spring solutions but also serves as a valuable reference model for other mechanical design challenges.

Consider

(15) $$x=\left[x_{1},\;x_{2},\;x_{3}\right]=\left[d,\;D,\;N\right],$$

Minimize

(16) $$f\left(\vec{x}\right)=\left(x_{3}+2\right)x_{2}x_{1}^{2},$$

Subject to

(17) $$\begin{array}{c} \begin{array}{c} g_{1}\left(\vec{x}\right)=1-\frac{x_{2}^{3}x_{3}}{71,755x_{1}^{4}}\leq 0\\ g_{2}\left(\vec{x}\right)=\frac{4x_{2}^{2}-x_{1}x_{2}}{12,566\left(x_{2}x_{1}^{3}-x_{1}^{4}\right)}+\frac{1}{5108x_{1}^{2}}\leq 0\\ g_{3}\left(\vec{x}\right)=1-\frac{140.45x_{1}}{x_{2}^{2}x_{3}}\leq 0\\ g_{4}\left(\vec{x}\right)=\frac{x_{1}+x_{2}}{1.5}-1\leq 0 \end{array} \end{array}$$

where $0.05\leq x_{1}\leq 2,\;0.25\leq x_{2}\leq 1.3,$ and $2\leq x_{3}\leq 15$.

**Figure 7:**
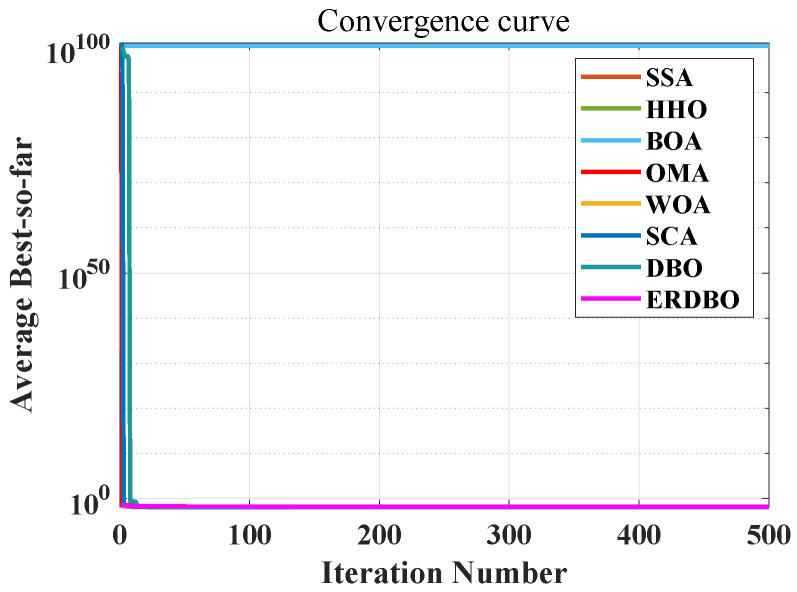
The tension (compression) spring-design convergence plot.

The convergence curve is shown in Figure 7. Table 7 summarizes the results obtained from 100 independent runs of the tension/compression spring-design optimization problem, comparing the performance of several state-of-the-art algorithms. Notably, the ERDBO algorithm consistently achieves the lowest objective function value across all the trials, demonstrating its superior capability in identifying high-quality solutions. This performance highlights the algorithm’s effectiveness in navigating the complex nonlinear design spaces inherent to spring optimization tasks.

The consistent dominance of the ERDBO in terms of solution quality suggests strong robustness and convergence reliability, even under repeated experimental conditions. Such performance is particularly valuable in engineering design applications, where stability across multiple executions is essential. These results reinforce the practical potential of the ERDBO in solving real-world design problems, where cost reduction and performance optimization are critical objectives. Consequently, the ERDBO stands out as a promising tool for advancing intelligent design methodologies in mechanical and structural system optimization.

### 5.2. Three-Bar Truss-Design Problem

The design of a three-pole truss constitutes a multifaceted engineering challenge that requires balancing structural integrity, material cost, and environmental impact. Unlike conventional architectural tasks, this problem [36,37] demands the integration of mechanics, materials science, and mathematical modeling.

Ensuring structural stability and safety necessitates precise stress and deformation analyses for each truss member under varying load conditions, with strict adherence to the design specifications. Simultaneously, minimizing the overall structural volume is critical for reducing material consumption and construction costs.

**Figure 8:**
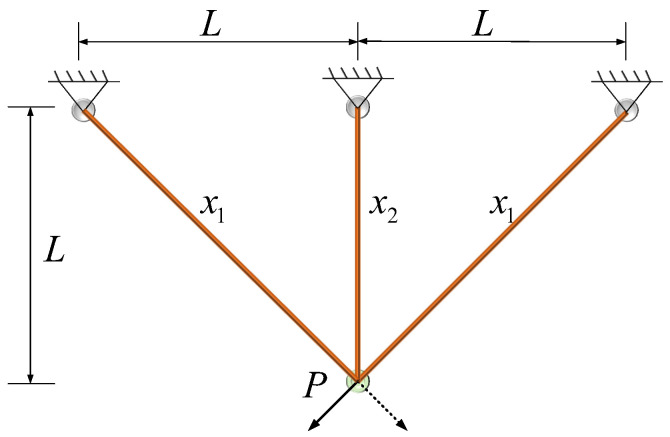
Triangle truss design.

To address this optimization problem, advanced mathematical techniques such as linear and nonlinear programming, alongside structural optimization methodologies, are employed. These approaches facilitate the identification of design solutions that achieve an effective trade-off between mechanical performance and material efficiency. The truss with three poles in the design is illustrated in Figure 8.

Given the growing emphasis on resource scarcity and environmental sustainability, such optimization efforts are increasingly significant. The development of efficient, lightweight, and sustainable truss structures not only enhances engineering performance but also contributes to long-term goals of resource conservation and ecological responsibility.

Consider variable

(18) $$\begin{array}{c} x=(x_{1},x_{2}). \end{array}$$

Minimize

(19) $$\begin{array}{c} \min f\left(x\right)=\left(2\sqrt{2}x_{1}+x_{2}\right)\times L. \end{array}$$

Subject to

(20) $$\begin{array}{cc} \; & g_{1}=\frac{\sqrt{2}x_{1}+x_{2}}{\sqrt{2}x_{1}^{2}+2x_{1}x_{2}}P-\sigma \leq 0\\ \; & g_{2}=\frac{x_{2}}{\sqrt{2}x_{1}^{2}+2x_{1}x_{2}}P-\sigma \leq 0\\ \; & g_{3}=\frac{1}{x_{1}+\sqrt{2}x_{2}}P-\sigma \leq 0 \end{array}$$

where P=2, L=100, σ=2.

With bounds $0\leq x_{1},\;x_{2}\leq 2$.

Recent advancements in metaheuristic algorithms have further enhanced the efficiency of truss optimization. Among these, the algorithm has demonstrated promising performance in solving high-dimensional nonlinear structural design problems. The ERDBO builds upon the standard DBO framework by incorporating adaptive refracted search strategies and dynamic perturbation mechanisms, which significantly improve global convergence and robustness against local optima.

In truss structure optimization, the ERDBO enables efficient exploration of the design space while maintaining exploitation precision, thereby ensuring that the final solution achieves both mechanical soundness and material economy. Comparative simulations against other state-of-the-art algorithms such as PSO, GA, and standard DBO indicate that the ERDBO achieves faster convergence rates and lower structural weights under identical constraint conditions (Table 8).

**Figure 9:**
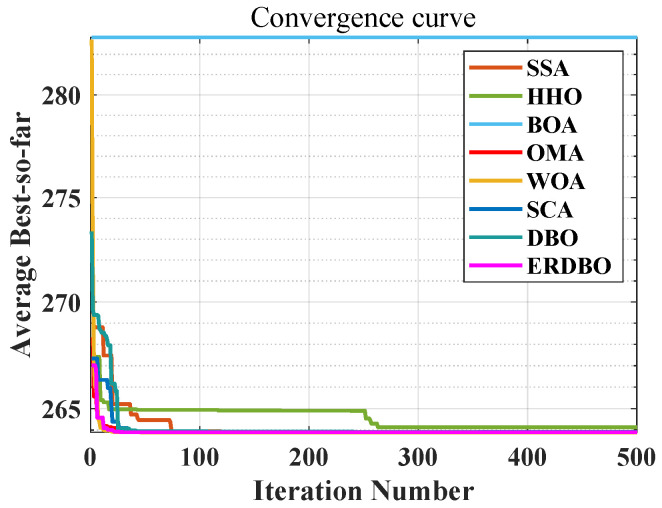
Three-bar truss convergence curve diagram.

The convergence curve is shown in Figure 9. Table 9 presents the comparison results. The application of the ERDBO to the three-pole truss design not only validates the algorithm’s effectiveness but also underscores its potential in addressing complex real-world structural engineering problems. Its flexibility and scalability make it a viable tool for broader optimization challenges in civil and mechanical engineering.

### 5.3. Pressure Vessel Problem

**Figure 10:**
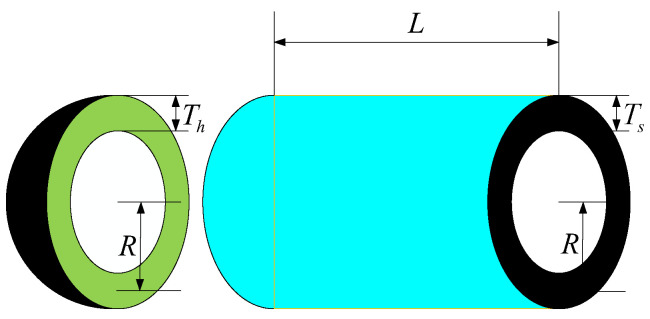
Pressure vessel parameter diagram.

The primary objective in pressure vessel [38] design is to achieve cost-effective manufacturing without compromising functional performance. This necessitates the multi-objective optimization of four critical design parameters: shell thickness (Ts), head thickness (Th), inner radius (R), and the cylindrical body length excluding the heads (L) (Figure 10). These parameters must be carefully coordinated to ensure the structural integrity, pressure-bearing capacity, and long-term durability of the vessel while minimizing material and fabrication costs.

Specifically, the thicknesses of the shell and heads are directly correlated with the vessel’s ability to withstand internal pressure and mechanical stresses. In contrast, the inner radius and cylindrical length predominantly influence the internal storage volume and surface area, which in turn affect material usage and manufacturing complexity. Therefore, achieving an optimal configuration of these variables requires a holistic approach that considers the mechanical properties of construction materials, fabrication techniques, regulatory safety codes, and economic constraints. Such a comprehensive design strategy is essential for developing efficient, reliable, and economically viable pressure vessels.

Consider variable

(21) $$\begin{array}{c} \vec{x}=\left[x_{1}\;x_{2}\;x_{3}\;x_{4}\right]=\left[T_{s}\;T_{h}\;R\;L\right] \end{array}$$

Minimize

(22) $$\begin{array}{c} f\left(\vec{x}\right)=0.6224x_{1}x_{3}x_{4}+1.7781x_{2}x_{3}^{2}+3.1661x_{1}^{2}x_{4}+19.84x_{1}^{2}x_{3} \end{array}$$

Subject to

(23) $$\begin{array}{c} \begin{array}{c} g_{1}\left(\vec{x}\right)=-x_{1}+0.0193x_{3}\\ g_{2}\left(\vec{x}\right)=-x_{3}+0.00954x_{3}\\ g_{3}\left(\vec{x}\right)=-\pi x_{3}^{2}x_{4}-\frac{4}{3}\pi x_{3}^{3}+12,960,000\\ g_{4}\left(\vec{x}\right)=x_{4}-2400 \end{array} \end{array}$$

Parameter range: $0⩽x_{1},\;x_{2}⩽99,\;10⩽x_{3},\;x_{4}⩽200.$

A comprehensive analysis of the pressure vessel model illustrated in Figure 9, along with the convergence behavior presented in the iteration curve of Figure 10, clearly demonstrates that the ERDBO algorithm achieves the optimal solution with significantly fewer iterations compared to its counterparts. This efficiency highlights the algorithm’s exceptional capability in addressing the pressure vessel design problem. The results not only affirm the effectiveness and robustness of the ERDBO algorithm in solving complex engineering optimization tasks but also offer meaningful contributions to both academic research and industrial practice within the domain of pressure vessel design.

**Figure 11:**
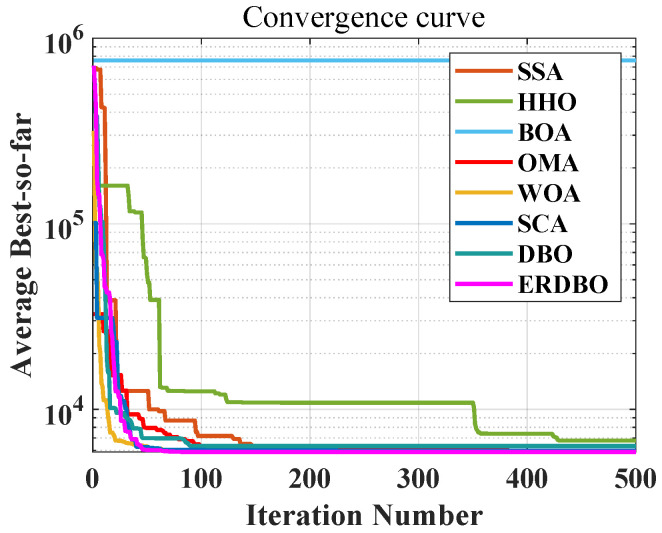
The iteration plot of the pressure vessel problem.

Moreover, the iterative performance depicted in Figure 11 further substantiates the superiority of the ERDBO algorithm over all other benchmark methods. Under identical problem settings, the ERDBO consistently yields better performance metrics, thereby validating its strong applicability in practical design scenarios. Through comparative analysis of algorithmic performance, this study provides critical insights into the relative advantages and limitations of various optimization approaches, offering a valuable foundation for advancing intelligent design strategies in pressure vessel engineering.

Table 9 presents a detailed statistical analysis based on 100 independent runs of the pressure vessel design optimization task. Among the eight algorithms evaluated, the ERDBO algorithm achieves the lowest mean objective function value, indicating superior optimization capability. Although the ERDBO exhibits marginally lower stability compared to the WOA algorithm, it still demonstrates greater consistency than the original DBO algorithm. This suggests that, with appropriate parameter settings, the ERDBO offers notable advantages in solving pressure vessel design problems.

When considering both average performance and result stability, the ERDBO consistently outperforms its peers, underscoring its effectiveness and robustness. These findings not only reinforce the potential of the ERDBO in addressing complex engineering optimization challenges but also provide a solid foundation for its application in real-world design processes. The statistical evidence further supports its adoption in practical scenarios and encourages future exploration in intelligent optimization for pressure vessel design.

## 6. Conclusions

This paper proposed the Enhanced Reproductive Dung Beetle Optimizer (ERDBO) and applied it to solving complex optimization problems. By incorporating random seeds and trigonometric functions derived from the dung beetle’s reproductive strategy, the ERDBO enhances the balance between global exploration and local exploitation, thereby improving both convergence speed and solution accuracy. On the 30-dimensional and 100-dimensional CEC2017 benchmark functions, the experimental results demonstrate that the ERDBO significantly outperforms several recent metaheuristic algorithms in terms of mean value, standard deviation, and robustness.

Furthermore, the applicability of the ERDBO was validated through classical engineering optimization cases, including the tension/compression spring-design problem, the three-bar truss design problem, and the pressure vessel problem. The results indicate that the ERDBO can achieve competitive or even superior solutions while maintaining computational efficiency. These findings highlight the reliability and potential of the ERDBO in both theoretical research and practical applications.

Future research will focus on extending the ERDBO to dynamic and large-scale optimization problems, as well as exploring its integration with machine learning and intelligent control frameworks to advance applications in power systems, smart grids, and sustainable engineering design.

## Figures and Tables

**Table 1:** CEC2017 functions.

Type	No.	Function	Minimum Value
Unimodal functions	1	Shifted and Rotated Bent Cigar Function	100
	2	Shifted and Rotated Zakharov Function	200
Simple multimodal functions	3	Shifted and Rotated Rosenbrock Function	300
	4	Shifted and Rotated Rastrigin Function	400
	5	Shifted and Rotated Expanded Scaffer’s F6 Function	500
	6	Shifted and Rotated Lunacek Bi_Rastrigin Function	600
	7	Shifted and Rotated Noncontinuous Rastrigin Function	700
	8	Shifted and Rotated Lévy Function	800
	9	Shifted and Rotated Schwefel Function	900
Hybrid functions	10	Hybrid Function 1 (N = 3)	1000
	11	Hybrid function 2 (N = 3)	1100
	12	Hybrid function 3 (N = 3)	1200
	13	Hybrid function 4 (N = 4)	1300
	14	Hybrid function 5 (N = 4)	1400
	15	Hybrid function 6 (N = 4)	1500
	16	Hybrid function 6 (N = 5)	1600
	17	Hybrid function 6 (N = 5)	1700
	18	Hybrid function 6 (N = 5)	1800
	19	Hybrid function 6 (N = 6)	1900
Composition functions	20	Composition Function 1 (N = 3)	2000
	21	Composition function 2 (N = 3)	2100
	22	Composition function 3 (N = 4)	2200
	23	Composition function 4 (N = 4)	2300
	24	Composition function 5 (N = 5)	2400
	25	Composition function 6 (N = 5)	2500
	26	Composition function 7 (N = 6)	2600
	27	Composition function 7 (N = 6)	2700
	28	Composition function 9 (N = 3)	2800
	29	Composition function 10 (N = 3)	2900
Search range: ${\left[-100,100\right]}^{D}$			

**Table 2:** Algorithm parameters.

Algorithm	Population	Number of Iterations	Parameters
SSA [15]	30	500	PD=0.2; SD=0.1; R=0.8
HHO [21]	30	500	β=1.5; r=0.5; E=0.5
BOA [14]	30	500	P=0.8; pe=0.1; sm=0.01
OMA	30	500	NA=1.40
WOA [20]	30	500	$a=2*(1-t/T_{\max});\;k=1$
SCA [18]	30	500	T=0
DBO [13]	30	500	RDB=6; EDB=6; FDB=7; *SDB* = 11
ERDBO	30	500	r=0.5

**Table 3:** CEC2017 test results for 30 dimensions.

		Dim = 30							
		SSA	HHO	BOA	OMA	WOA	SCA	DBO	ERDBO
F1	min	1.12 $\times \;10^{2}$	1.61 $\times \;10^{8}$	5.14 $\times \;10^{10}$	3.35 $\times \;10^{8}$	1.54 $\times \;10^{6}$	1.40 $\times \;10^{10}$	7.65 $\times \;10^{4}$	4.10 $\times \;10^{4}$
	mean	**4.80** $\;\times \;10^{3}$	4.31 $\times \;10^{8}$	7.71 $\times \;10^{10}$	2.28 $\times \;10^{9}$	4.27 $\times \;10^{8}$	2.07 $\times \;10^{10}$	2.29 $\times \;10^{8}$	6.33 $\times \;10^{4}$
	std	2.76 $\times \;10^{7}$	6.25 $\times \;10^{16}$	7.19 $\times \;10^{19}$	4.53 $\times \;10^{18}$	6.28 $\times \;10^{17}$	1.64 $\times \;10^{19}$	1.68 $\times \;10^{16}$	2.34 $\times \;10^{14}$
	degree	1	4	8	6	5	7	3	2
F2	min	3.28 $\times \;10^{4}$	3.43 $\times \;10^{4}$	7.72 $\times \;10^{4}$	4.03 $\times \;10^{4}$	4.78 $\times \;10^{3}$	6.15 $\times \;10^{4}$	5.99 $\times \;10^{4}$	3.47 $\times \;10^{4}$
	mean	4.64 $\times \;10^{4}$	5.78 $\times \;10^{4}$	8.61 $\times \;10^{5}$	6.35 $\times \;10^{4}$	**1.33** $\;\times \;10^{4}$	8.70 $\times \;10^{4}$	9.14 $\times \;10^{4}$	6.24 $\times \;10^{4}$
	std	3.69 $\times \;10^{7}$	5.81 $\times \;10^{7}$	1.08 $\times \;10^{13}$	1.98 $\times \;10^{8}$	2.44 $\times \;10^{7}$	3.03 $\times \;10^{8}$	3.45 $\times \;10^{8}$	1.91 $\times \;10^{8}$
	degree	2	3	8	5	1	6	7	4
F3	min	4.73 $\times \;10^{2}$	5.85 $\times \;10^{2}$	6.14 $\times \;10^{3}$	5.91 $\times \;10^{2}$	4.81 $\times \;10^{2}$	1.69 $\times \;10^{3}$	5.19 $\times \;10^{2}$	4.26 $\times \;10^{2}$
	mean	5.02 $\times \;10^{2}$	7.41 $\times \;10^{2}$	1.64 $\times \;10^{4}$	8.01 $\times \;10^{2}$	5.79 $\times \;10^{2}$	2.72 $\times \;10^{3}$	6.39 $\times \;10^{2}$	**4.53** $\;\times \;10^{2}$
	std	4.61 $\times \;10^{2}$	1.44 $\times \;10^{4}$	3.58 $\times \;10^{7}$	2.34 $\times \;10^{4}$	2.62 $\times \;10^{3}$	6.08 $\times \;10^{5}$	5.50 $\times \;10^{3}$	2.69 $\times \;10^{3}$
	degree	2	5	8	6	3	7	4	1
F4	min	6.33 $\times \;10^{2}$	7.10 $\times \;10^{2}$	8.70 $\times \;10^{2}$	6.22 $\times \;10^{2}$	6.38 $\times \;10^{2}$	7.85 $\times \;10^{2}$	6.61 $\times \;10^{2}$	6.01 $\times \;10^{2}$
	mean	7.51 $\times \;10^{2}$	7.73 $\times \;10^{2}$	9.71 $\times \;10^{2}$	7.15 $\times \;10^{2}$	6.99 $\times \;10^{2}$	8.25 $\times \;10^{2}$	7.47 $\times \;10^{2}$	**6.96** $\;\times \;10^{2}$
	std	2.61 $\times \;10^{3}$	6.05 $\times \;10^{2}$	2.07 $\times \;10^{3}$	1.61 $\times \;10^{3}$	1.07 $\times \;10^{3}$	7.21 $\times \;10^{2}$	1.68 $\times \;10^{3}$	2.53 $\times \;10^{3}$
	degree	5	6	8	3	2	7	4	1
F5	min	6.25 $\times \;10^{2}$	6.61 $\times \;10^{2}$	6.73 $\times \;10^{2}$	6.12 $\times \;10^{2}$	6.38 $\times \;10^{2}$	6.48 $\times \;10^{2}$	6.34 $\times \;10^{2}$	6.18 $\times \;10^{2}$
	mean	6.52 $\times \;10^{2}$	6.74 $\times \;10^{2}$	7.01 $\times \;10^{2}$	6.36 $\times \;10^{2}$	6.45 $\times \;10^{2}$	6.70 $\times \;10^{2}$	6.58 $\times \;10^{2}$	**6.28** $\;\times \;10^{2}$
	std	1.90 $\times \;10^{2}$	4.12 $\times \;10^{1}$	2.13 $\times \;10^{2}$	8.06 $\times \;10^{1}$	6.44 $\times \;10^{1}$	4.79 $\times \;10^{1}$	1.14 $\times \;10^{2}$	1.32 $\times \;10^{2}$
	degree	3	7	8	2	4	6	5	1
F6	min	1.05 $\times \;10^{3}$	1.18 $\times \;10^{3}$	1.38 $\times \;10^{3}$	1.02 $\times \;10^{3}$	9.45 $\times \;10^{2}$	1.15 $\times \;10^{3}$	8.72 $\times \;10^{2}$	9.05 $\times \;10^{2}$
	mean	1.21 $\times \;10^{3}$	1.34 $\times \;10^{3}$	1.52 $\times \;10^{3}$	1.16 $\times \;10^{3}$	1.08 $\times \;10^{3}$	1.27 $\times \;10^{3}$	1.04 $\times \;10^{3}$	**9.65** $\;\times \;10^{2}$
	std	9.82 $\times \;10^{3}$	4.95 $\times \;10^{3}$	5.74 $\times \;10^{3}$	7.85 $\times \;10^{3}$	6.98 $\times \;10^{3}$	7.22 $\times \;10^{3}$	6.15 $\times \;10^{3}$	5.54 $\times \;10^{3}$
	degree	5	7	8	4	3	6	2	1
F7	min	9.44 $\times \;10^{2}$	9.68 $\times \;10^{2}$	1.17 $\times \;10^{3}$	9.29 $\times \;10^{2}$	8.85 $\times \;10^{2}$	1.08 $\times \;10^{3}$	9.18 $\times \;10^{2}$	8.75 $\times \;10^{2}$
	mean	9.74 $\times \;10^{2}$	9.95 $\times \;10^{2}$	1.24 $\times \;10^{3}$	9.72 $\times \;10^{2}$	9.47 $\times \;10^{2}$	1.12 $\times \;10^{3}$	1.04 $\times \;10^{3}$	**9.16** $\;\times \;10^{2}$
	std	1.10 $\times \;10^{3}$	2.13 $\times \;10^{2}$	1.58 $\times \;10^{3}$	6.15 $\times \;10^{2}$	4.40 $\times \;10^{2}$	4.68 $\times \;10^{2}$	2.50 $\times \;10^{3}$	1.37 $\times \;10^{3}$
	degree	3	5	8	4	2	7	6	1
F8	min	3.79 $\times \;10^{3}$	6.70 $\times \;10^{3}$	1.34 $\times \;10^{4}$	1.82 $\times \;10^{3}$	1.89 $\times \;10^{3}$	5.17 $\times \;10^{3}$	2.88 $\times \;10^{3}$	2.46 $\times \;10^{3}$
	mean	5.30 $\times \;10^{3}$	8.45 $\times \;10^{3}$	1.69 $\times \;10^{4}$	3.78 $\times \;10^{3}$	4.08 $\times \;10^{3}$	8.58 $\times \;10^{3}$	7.29 $\times \;10^{3}$	**3.57** $\;\times \;10^{3}$
	std	1.57 $\times \;10^{5}$	1.16 $\times \;10^{6}$	3.36 $\times \;10^{6}$	1.23 $\times \;10^{6}$	7.41 $\times \;10^{5}$	3.22 $\times \;10^{6}$	6.94 $\times \;10^{6}$	2.30 $\times \;10^{6}$
	degree	4	6	8	2	3	7	5	1
F9	min	3.61 $\times \;10^{3}$	4.92 $\times \;10^{3}$	9.18 $\times \;10^{3}$	6.68 $\times \;10^{3}$	3.71 $\times \;10^{3}$	8.10 $\times \;10^{3}$	4.62 $\times \;10^{3}$	3.69 $\times \;10^{3}$
	mean	5.44 $\times \;10^{3}$	6.41 $\times \;10^{3}$	1.05 $\times \;10^{4}$	8.39 $\times \;10^{3}$	5.18 $\times \;10^{3}$	8.75 $\times \;10^{3}$	6.63 $\times \;10^{3}$	**4.21** $\;\times \;10^{3}$
	std	6.67 $\times \;10^{5}$	4.43 $\times \;10^{5}$	1.81 $\times \;10^{5}$	4.05 $\times \;10^{5}$	9.35 $\times \;10^{5}$	1.35 $\times \;10^{5}$	1.36 $\times \;10^{6}$	1.10 $\times \;10^{6}$
	degree	3	4	8	6	2	7	5	1
F10	min	1.18 $\times \;10^{3}$	1.34 $\times \;10^{3}$	5.60 $\times \;10^{3}$	1.23 $\times \;10^{3}$	1.18 $\times \;10^{3}$	2.29 $\times \;10^{3}$	1.33 $\times \;10^{3}$	1.15 $\times \;10^{3}$
	mean	1.31 $\times \;10^{3}$	1.60 $\times \;10^{3}$	2.59 $\times \;10^{4}$	1.39 $\times \;10^{3}$	**1.29** $\;\times \;10^{3}$	3.65 $\times \;10^{3}$	1.89 $\times \;10^{3}$	1.37 $\times \;10^{3}$
	std	5.33 $\times \;10^{3}$	5.25 $\times \;10^{4}$	1.69 $\times \;10^{8}$	7.59 $\times \;10^{3}$	6.81 $\times \;10^{3}$	1.06 $\times \;10^{6}$	2.94 $\times \;10^{5}$	6.59 $\times \;10^{3}$
	degree	2	5	8	4	1	7	6	3
F11	min	3.08 $\times \;10^{4}$	1.14 $\times \;10^{7}$	1.07 $\times \;10^{10}$	2.03 $\times \;10^{6}$	2.19 $\times \;10^{5}$	1.54 $\times \;10^{9}$	1.63 $\times \;10^{6}$	1.40 $\times \;10^{5}$
	mean	**1.19** $\;\times \;10^{6}$	8.88 $\times \;10^{7}$	2.08 $\times \;10^{10}$	2.10 $\times \;10^{7}$	2.06 $\times \;10^{6}$	2.81 $\times \;10^{9}$	5.68 $\times \;10^{7}$	8.68 $\times \;10^{6}$
	std	7.79 $\times \;10^{11}$	5.87 $\times \;10^{15}$	2.94 $\times \;10^{19}$	4.20 $\times \;10^{14}$	3.89 $\times \;10^{12}$	5.71 $\times \;10^{17}$	6.29 $\times \;10^{15}$	1.46 $\times \;10^{14}$
	degree	1	4	8	6	5	7	2	3
F12	min	3.20 $\times \;10^{3}$	4.80 $\times \;10^{5}$	4.40 $\times \;10^{9}$	1.20 $\times \;10^{4}$	5.10 $\times \;10^{3}$	5.80 $\times \;10^{8}$	2.50 $\times \;10^{4}$	7.50 $\times \;10^{3}$
	mean	3.25 $\times \;10^{4}$	1.38 $\times \;10^{6}$	1.90 $\times \;10^{10}$	2.35 $\times \;10^{5}$	**1.85** $\;\times \;10^{4}$	1.08 $\times \;10^{9}$	5.05 $\times \;10^{6}$	1.97 $\times \;10^{5}$
	std	8.10 $\times \;10^{8}$	1.34 $\times \;10^{12}$	5.60 $\times \;10^{19}$	5.40 $\times \;10^{11}$	2.60 $\times \;10^{8}$	9.30 $\times \;10^{16}$	1.04 $\times \;10^{14}$	3.85 $\times \;10^{11}$
	degree	2	5	8	4	1	7	6	3
F13	min	9.85 $\times \;10^{3}$	3.90 $\times \;10^{4}$	2.50 $\times \;10^{6}$	2.60 $\times \;10^{3}$	1.65 $\times \;10^{3}$	8.40 $\times \;10^{4}$	6.50 $\times \;10^{3}$	2.35 $\times \;10^{3}$
	mean	6.55 $\times \;10^{4}$	1.47 $\times \;10^{6}$	2.25 $\times \;10^{7}$	3.70 $\times \;10^{4}$	**5.10** $\;\times \;10^{3}$	8.80 $\times \;10^{5}$	4.75 $\times \;10^{5}$	2.60 $\times \;10^{5}$
	std	1.65 $\times \;10^{9}$	1.60 $\times \;10^{12}$	2.28 $\times \;10^{14}$	1.85 $\times \;10^{9}$	4.80 $\times \;10^{7}$	4.10 $\times \;10^{11}$	1.15 $\times \;10^{12}$	6.10 $\times \;10^{10}$
	degree	3	7	8	2	1	6	5	4
F14	min	2.20 $\times \;10^{3}$	3.70 $\times \;10^{4}$	5.25 $\times \;10^{8}$	2.25 $\times \;10^{3}$	1.90 $\times \;10^{3}$	2.05 $\times \;10^{6}$	1.10 $\times \;10^{4}$	2.00 $\times \;10^{3}$
	mean	1.30 $\times \;10^{4}$	1.28 $\times \;10^{5}$	3.25 $\times \;10^{9}$	9.60 $\times \;10^{3}$	**4.20** $\;\times \;10^{3}$	6.70 $\times \;10^{7}$	1.20 $\times \;10^{5}$	1.25 $\times \;10^{4}$
	std	1.28 $\times \;10^{8}$	4.70 $\times \;10^{9}$	2.85 $\times \;10^{18}$	8.85 $\times \;10^{7}$	6.90 $\times \;10^{6}$	2.40 $\times \;10^{15}$	4.70 $\times \;10^{10}$	1.38 $\times \;10^{8}$
	degree	4	6	8	2	1	7	5	3
F15	min	2.20 $\times \;10^{3}$	2.80 $\times \;10^{3}$	4.15 $\times \;10^{3}$	2.75 $\times \;10^{3}$	2.18 $\times \;10^{3}$	3.55 $\times \;10^{3}$	2.28 $\times \;10^{3}$	1.82 $\times \;10^{3}$
	mean	2.95 $\times \;10^{3}$	3.60 $\times \;10^{3}$	5.55 $\times \;10^{3}$	3.42 $\times \;10^{3}$	2.78 $\times \;10^{3}$	4.25 $\times \;10^{3}$	3.30 $\times \;10^{3}$	**2.63** $\;\times \;10^{3}$
	std	6.95 $\times \;10^{4}$	2.35 $\times \;10^{5}$	6.05 $\times \;10^{5}$	9.50 $\times \;10^{4}$	9.45 $\times \;10^{4}$	9.00 $\times \;10^{4}$	1.78 $\times \;10^{5}$	6.80 $\times \;10^{4}$
	degree	3	6	8	5	2	7	4	1
F16	min	1.88 $\times \;10^{3}$	2.20 $\times \;10^{3}$	2.95 $\times \;10^{3}$	1.98 $\times \;10^{3}$	1.92 $\times \;10^{3}$	2.11 $\times \;10^{3}$	2.14 $\times \;10^{3}$	1.72 $\times \;10^{3}$
	mean	2.39 $\times \;10^{3}$	2.70 $\times \;10^{3}$	3.90 $\times \;10^{3}$	**2.25** $\;\times \;10^{3}$	2.45 $\times \;10^{3}$	2.85 $\times \;10^{3}$	2.65 $\times \;10^{3}$	2.60 $\times \;10^{3}$
	std	6.60 $\times \;10^{4}$	1.07 $\times \;10^{5}$	2.25 $\times \;10^{5}$	2.70 $\times \;10^{4}$	8.70 $\times \;10^{4}$	7.00 $\times \;10^{4}$	5.60 $\times \;10^{4}$	9.60 $\times \;10^{4}$
	degree	2	6	8	1	3	7	5	4
F17	min	5.12 $\times \;10^{4}$	8.70 $\times \;10^{4}$	5.60 $\times \;10^{7}$	7.30 $\times \;10^{4}$	1.44 $\times \;10^{4}$	2.77 $\times \;10^{6}$	1.42 $\times \;10^{5}$	2.35 $\times \;10^{4}$
	mean	7.15 $\times \;10^{5}$	4.80 $\times \;10^{6}$	4.30 $\times \;10^{8}$	4.20 $\times \;10^{5}$	**1.15** $\;\times \;10^{5}$	1.20 $\times \;10^{7}$	3.30 $\times \;10^{6}$	1.25 $\times \;10^{6}$
	std	8.70 $\times \;10^{11}$	3.92 $\times \;10^{13}$	8.95 $\times \;10^{16}$	2.50 $\times \;10^{11}$	1.40 $\times \;10^{10}$	4.70 $\times \;10^{13}$	6.15 $\times \;10^{13}$	4.25 $\times \;10^{12}$
	degree	3	6	8	2	1	7	5	4
F18	min	2.07 $\times \;10^{3}$	1.12 $\times \;10^{5}$	9.30 $\times \;10^{8}$	2.75 $\times \;10^{3}$	2.08 $\times \;10^{3}$	2.65 $\times \;10^{7}$	4.80 $\times \;10^{3}$	2.10 $\times \;10^{3}$
	mean	1.44 $\times \;10^{4}$	1.70 $\times \;10^{6}$	3.65 $\times \;10^{9}$	1.59 $\times \;10^{4}$	**7.20** $\;\times \;10^{3}$	8.55 $\times \;10^{7}$	1.80 $\times \;10^{7}$	1.23 $\times \;10^{4}$
	std	2.05 $\times \;10^{8}$	2.33 $\times \;10^{12}$	3.40 $\times \;10^{18}$	1.30 $\times \;10^{8}$	2.85 $\times \;10^{7}$	2.26 $\times \;10^{15}$	4.35 $\times \;10^{15}$	1.42 $\times \;10^{8}$
	degree	3	5	8	4	1	7	6	2
F19	min	2.48 $\times \;10^{3}$	2.44 $\times \;10^{3}$	3.05 $\times \;10^{3}$	2.45 $\times \;10^{3}$	2.29 $\times \;10^{3}$	2.57 $\times \;10^{3}$	2.51 $\times \;10^{3}$	2.05 $\times \;10^{3}$
	mean	2.77 $\times \;10^{3}$	2.91 $\times \;10^{3}$	3.59 $\times \;10^{3}$	2.66 $\times \;10^{3}$	2.54 $\times \;10^{3}$	2.97 $\times \;10^{3}$	2.83 $\times \;10^{3}$	**2.43** $\;\times \;10^{3}$
	std	3.86 $\times \;10^{4}$	6.53 $\times \;10^{4}$	4.88 $\times \;10^{4}$	1.33 $\times \;10^{4}$	2.07 $\times \;10^{4}$	2.50 $\times \;10^{4}$	2.45 $\times \;10^{4}$	5.09 $\times \;10^{4}$
	degree	4	6	8	3	2	7	5	1
F20	min	2.43 $\times \;10^{3}$	2.47 $\times \;10^{3}$	2.60 $\times \;10^{3}$	2.42 $\times \;10^{3}$	2.39 $\times \;10^{3}$	2.58 $\times \;10^{3}$	2.36 $\times \;10^{3}$	2.20 $\times \;10^{3}$
	mean	2.52 $\times \;10^{3}$	2.59 $\times \;10^{3}$	2.78 $\times \;10^{3}$	2.50 $\times \;10^{3}$	2.48 $\times \;10^{3}$	2.63 $\times \;10^{3}$	2.56 $\times \;10^{3}$	**2.48** $\;\times \;10^{3}$
	std	2.56 $\times \;10^{3}$	2.76 $\times \;10^{3}$	4.47 $\times \;10^{3}$	1.02 $\times \;10^{3}$	2.09 $\times \;10^{3}$	7.60 $\times \;10^{2}$	4.30 $\times \;10^{3}$	4.32 $\times \;10^{3}$
	degree	4	6	8	3	2	7	5	1
F21	min	2.31 $\times \;10^{3}$	2.76 $\times \;10^{3}$	6.69 $\times \;10^{3}$	2.47 $\times \;10^{3}$	2.33 $\times \;10^{3}$	3.95 $\times \;10^{3}$	2.40 $\times \;10^{3}$	2.23 $\times \;10^{3}$
	mean	5.51 $\times \;10^{3}$	7.70 $\times \;10^{3}$	1.12 $\times \;10^{4}$	**2.77** $\;\times \;10^{3}$	2.97 $\times \;10^{3}$	9.94 $\times \;10^{3}$	5.02 $\times \;10^{3}$	2.99 $\times \;10^{3}$
	std	4.91 $\times \;10^{6}$	1.20 $\times \;10^{6}$	1.36 $\times \;10^{6}$	4.90 $\times \;10^{4}$	1.49 $\times \;10^{6}$	2.42 $\times \;10^{6}$	5.53 $\times \;10^{6}$	2.29 $\times \;10^{6}$
	degree	5	6	8	1	2	7	4	3
F22	min	2.76 $\times \;10^{3}$	3.03 $\times \;10^{3}$	3.09 $\times \;10^{3}$	2.82 $\times \;10^{3}$	2.82 $\times \;10^{3}$	2.99 $\times \;10^{3}$	2.82 $\times \;10^{3}$	2.54 $\times \;10^{3}$
	mean	2.91 $\times \;10^{3}$	3.30 $\times \;10^{3}$	3.42 $\times \;10^{3}$	**2.90** $\;\times \;10^{3}$	2.95 $\times \;10^{3}$	3.07 $\times \;10^{3}$	3.03 $\times \;10^{3}$	2.97 $\times \;10^{3}$
	std	8.61 $\times \;10^{3}$	2.38 $\times \;10^{4}$	2.36 $\times \;10^{4}$	1.83 $\times \;10^{3}$	6.68 $\times \;10^{3}$	2.37 $\times \;10^{3}$	6.73 $\times \;10^{3}$	5.91 $\times \;10^{3}$
	degree	2	7	8	1	3	6	5	4
F23	min	2.92 $\times \;10^{3}$	3.27 $\times \;10^{3}$	3.25 $\times \;10^{3}$	2.99 $\times \;10^{3}$	2.93 $\times \;10^{3}$	3.17 $\times \;10^{3}$	3.00 $\times \;10^{3}$	2.82 $\times \;10^{3}$
	mean	3.05 $\times \;10^{3}$	3.52 $\times \;10^{3}$	3.50 $\times \;10^{3}$	3.10 $\times \;10^{3}$	3.11 $\times \;10^{3}$	3.26 $\times \;10^{3}$	3.18 $\times \;10^{3}$	**2.79** $\;\times \;10^{3}$
	std	6.07 $\times \;10^{3}$	2.42 $\times \;10^{4}$	3.39 $\times \;10^{4}$	2.86 $\times \;10^{3}$	7.32 $\times \;10^{3}$	1.24 $\times \;10^{3}$	1.06 $\times \;10^{4}$	9.22 $\times \;10^{3}$
	degree	2	8	7	3	4	6	5	1
F24	min	2.88 $\times \;10^{3}$	2.95 $\times \;10^{3}$	4.23 $\times \;10^{3}$	3.00 $\times \;10^{3}$	2.90 $\times \;10^{3}$	3.29 $\times \;10^{3}$	2.89 $\times \;10^{3}$	2.61 $\times \;10^{3}$
	mean	**2.91** $\;\times \;10^{3}$	2.99 $\times \;10^{3}$	5.76 $\times \;10^{3}$	3.05 $\times \;10^{3}$	2.97 $\times \;10^{3}$	3.50 $\times \;10^{3}$	3.02 $\times \;10^{3}$	2.95 $\times \;10^{3}$
	std	1.94 $\times \;10^{2}$	1.02 $\times \;10^{3}$	5.99 $\times \;10^{5}$	3.45 $\times \;10^{3}$	1.22 $\times \;10^{3}$	3.85 $\times \;10^{4}$	4.56 $\times \;10^{3}$	1.15 $\times \;10^{3}$
	degree	1	5	8	6	4	7	3	2
F25	min	5.12 $\times \;10^{3}$	3.92 $\times \;10^{3}$	9.34 $\times \;10^{3}$	5.43 $\times \;10^{3}$	3.37 $\times \;10^{3}$	7.22 $\times \;10^{3}$	5.38 $\times \;10^{3}$	3.38 $\times \;10^{3}$
	mean	6.52 $\times \;10^{3}$	7.86 $\times \;10^{3}$	1.16 $\times \;10^{4}$	6.39 $\times \;10^{3}$	6.65 $\times \;10^{3}$	7.88 $\times \;10^{3}$	7.20 $\times \;10^{3}$	**6.26** $\;\times \;10^{3}$
	std	5.95 $\times \;10^{5}$	1.79 $\times \;10^{6}$	1.11 $\times \;10^{6}$	2.08 $\times \;10^{5}$	1.83 $\times \;10^{6}$	2.83 $\times \;10^{5}$	4.72 $\times \;10^{5}$	1.17 $\times \;10^{6}$
	degree	7	4	8	6	3	5	2	1
F26	min	3.22 $\times \;10^{3}$	3.28 $\times \;10^{3}$	3.57 $\times \;10^{3}$	3.28 $\times \;10^{3}$	3.23 $\times \;10^{3}$	3.41 $\times \;10^{3}$	3.25 $\times \;10^{3}$	3.10 $\times \;10^{3}$
	mean	3.26 $\times \;10^{3}$	3.65 $\times \;10^{3}$	4.11 $\times \;10^{3}$	3.34 $\times \;10^{3}$	3.39 $\times \;10^{3}$	3.57 $\times \;10^{3}$	3.33 $\times \;10^{3}$	3.30 $\times \;10^{3}$
	std	1.58 $\times \;10^{3}$	3.70 $\times \;10^{4}$	1.40 $\times \;10^{5}$	1.45 $\times \;10^{3}$	7.90 $\times \;10^{3}$	9.10 $\times \;10^{3}$	3.29 $\times \;10^{3}$	6.07 $\times \;10^{3}$
	degree	1	3	8	7	6	5	4	2
F27	min	3.20 $\times \;10^{3}$	3.32 $\times \;10^{3}$	5.79 $\times \;10^{3}$	3.38 $\times \;10^{3}$	3.24 $\times \;10^{3}$	3.94 $\times \;10^{3}$	3.29 $\times \;10^{3}$	3.29 $\times \;10^{3}$
	mean	**3.23** $\;\times \;10^{3}$	3.50 $\times \;10^{3}$	7.26 $\times \;10^{3}$	3.54 $\times \;10^{3}$	3.36 $\times \;10^{3}$	4.44 $\times \;10^{3}$	3.54 $\times \;10^{3}$	3.39 $\times \;10^{3}$
	std	5.12 $\times \;10^{2}$	1.11 $\times \;10^{4}$	6.85 $\times \;10^{5}$	1.21 $\times \;10^{4}$	2.25 $\times \;10^{3}$	8.10 $\times \;10^{4}$	1.48 $\times \;10^{5}$	2.82 $\times \;10^{3}$
	degree	1	7	8	6	3	5	4	2
F28	min	3.45 $\times \;10^{3}$	4.37 $\times \;10^{3}$	5.52 $\times \;10^{3}$	3.84 $\times \;10^{3}$	3.96 $\times \;10^{3}$	4.50 $\times \;10^{3}$	3.82 $\times \;10^{3}$	3.49 $\times \;10^{3}$
	mean	**4.15** $\;\times \;10^{3}$	5.02 $\times \;10^{3}$	7.39 $\times \;10^{3}$	4.23 $\times \;10^{3}$	4.45 $\times \;10^{3}$	5.15 $\times \;10^{3}$	4.44 $\times \;10^{3}$	4.43 $\times \;10^{3}$
	std	1.30 $\times \;10^{5}$	2.10 $\times \;10^{5}$	9.70 $\times \;10^{5}$	3.80 $\times \;10^{4}$	6.01 $\times \;10^{4}$	1.11 $\times \;10^{5}$	1.48 $\times \;10^{5}$	2.07 $\times \;10^{5}$
	degree	1	3	8	2	4	6	5	7
F29	min	5.67 $\times \;10^{3}$	6.23 $\times \;10^{5}$	8.12 $\times \;10^{8}$	3.67 $\times \;10^{4}$	6.03 $\times \;10^{3}$	8.43 $\times \;10^{7}$	2.18 $\times \;10^{4}$	1.98 $\times \;10^{4}$
	mean	**1.74** $\;\times \;10^{4}$	1.30 $\times \;10^{7}$	2.75 $\times \;10^{9}$	2.68 $\times \;10^{5}$	3.90 $\times \;10^{4}$	1.98 $\times \;10^{8}$	3.86 $\times \;10^{6}$	2.78 $\times \;10^{5}$
	std	9.53 $\times \;10^{7}$	2.22 $\times \;10^{14}$	2.32 $\times \;10^{18}$	5.97 $\times \;10^{10}$	2.95 $\times \;10^{9}$	5.67 $\times \;10^{15}$	1.84 $\times \;10^{13}$	5.10 $\times \;10^{11}$
	degree	1	3	8	4	2	7	6	5

**Table 4:** CEC2017 test results for 100 dimensions.

		Dim = 100							
		SSA	HHO	BOA	OMA	WOA	SCA	DBO	ERDBO
F1	min	1.83 $\times \;10^{8}$	3.14 $\times \;10^{10}$	2.75 $\times \;10^{11}$	7.68 $\times \;10^{10}$	2.70 $\times \;10^{10}$	1.87 $\times \;10^{11}$	2.24 $\times \;10^{10}$	1.89 $\times \;10^{8}$
	mean	**3.82** $\;\times \;10^{8}$	4.93 $\times \;10^{10}$	2.96 $\times \;10^{11}$	1.19 $\times \;10^{11}$	6.49 $\times \;10^{10}$	2.16 $\times \;10^{11}$	7.90 $\times \;10^{10}$	3.99 $\times \;10^{9}$
	std	1.06 $\times \;10^{16}$	6.48 $\times \;10^{19}$	2.19 $\times \;10^{19}$	4.77 $\times \;10^{20}$	1.96 $\times \;10^{20}$	2.37 $\times \;10^{20}$	4.79 $\times \;10^{21}$	1.33 $\times \;10^{20}$
	degree	1	3	8	6	4	7	5	2
F2	min	3.48 $\times \;10^{5}$	3.15 $\times \;10^{5}$	8.62 $\times \;10^{5}$	3.56 $\times \;10^{5}$	1.75 $\times \;10^{5}$	4.74 $\times \;10^{5}$	3.56 $\times \;10^{5}$	3.13 $\times \;10^{5}$
	mean	7.39 $\times \;10^{5}$	3.59 $\times \;10^{5}$	1.90 $\times \;10^{10}$	4.20 $\times \;10^{5}$	**2.44** $\;\times \;10^{5}$	6.05 $\times \;10^{5}$	7.79 $\times \;10^{5}$	4.02 $\times \;10^{5}$
	std	1.44 $\times \;10^{10}$	6.10 $\times \;10^{9}$	3.32E+21	1.12 $\times \;10^{9}$	4.20 $\times \;10^{8}$	5.70 $\times \;10^{9}$	9.74 $\times \;10^{10}$	1.25 $\times \;10^{10}$
	degree	6	2	8	4	1	5	7	3
F3	min	8.59 $\times \;10^{2}$	6.66 $\times \;10^{3}$	9.04 $\times \;10^{4}$	1.06 $\times \;10^{4}$	2.01 $\times \;10^{3}$	3.79 $\times \;10^{4}$	3.75 $\times \;10^{3}$	1.01 $\times \;10^{3}$
	mean	**1.00** $\;\times \;10^{3}$	9.00 $\times \;10^{3}$	1.19 $\times \;10^{5}$	1.75 $\times \;10^{4}$	7.48 $\times \;10^{3}$	5.13 $\times \;10^{4}$	1.57 $\times \;10^{4}$	1.17 $\times \;10^{3}$
	std	9.20 $\times \;10^{3}$	1.61 $\times \;10^{6}$	1.60 $\times \;10^{8}$	1.75 $\times \;10^{7}$	9.10 $\times \;10^{6}$	4.63 $\times \;10^{7}$	3.01 $\times \;10^{8}$	2.86 $\times \;10^{6}$
	degree	1	4	8	6	3	7	5	2
F4	min	1.29 $\times \;10^{3}$	1.56 $\times \;10^{3}$	2.20 $\times \;10^{3}$	1.46 $\times \;10^{3}$	1.33 $\times \;10^{3}$	1.97 $\times \;10^{3}$	1.19 $\times \;10^{3}$	1.14 $\times \;10^{3}$
	mean	1.38 $\times \;10^{3}$	1.67 $\times \;10^{3}$	2.29 $\times \;10^{3}$	1.73 $\times \;10^{3}$	1.47 $\times \;10^{3}$	2.06 $\times \;10^{3}$	1.71 $\times \;10^{3}$	**1.31** $\;\times \;10^{3}$
	std	1.80 $\times \;10^{3}$	2.68 $\times \;10^{3}$	3.31 $\times \;10^{3}$	1.33 $\times \;10^{4}$	3.97 $\times \;10^{3}$	3.15 $\times \;10^{3}$	5.43 $\times \;10^{4}$	5.50 $\times \;10^{3}$
	degree	2	4	8	6	3	7	5	1
F5	min	6.62 $\times \;10^{2}$	6.85 $\times \;10^{2}$	7.09 $\times \;10^{2}$	6.72 $\times \;10^{2}$	6.64 $\times \;10^{2}$	6.95 $\times \;10^{2}$	6.62 $\times \;10^{2}$	6.07 $\times \;10^{2}$
	mean	6.66 $\times \;10^{2}$	6.90 $\times \;10^{2}$	7.28 $\times \;10^{2}$	6.86 $\times \;10^{2}$	6.70 $\times \;10^{2}$	7.03 $\times \;10^{2}$	6.78 $\times \;10^{2}$	**6.50** $\;\times \;10^{2}$
	std	7.00 $\times \;10^{0}$	1.85 $\times \;10^{1}$	7.52 $\times \;10^{1}$	6.21 $\times \;10^{1}$	1.14 $\times \;10^{1}$	1.06 $\times \;10^{1}$	1.15 $\times \;10^{2}$	3.20 $\times \;10^{1}$
	degree	2	6	8	5	3	7	4	1
F6	min	2.58 $\times \;10^{3}$	3.46 $\times \;10^{3}$	4.05 $\times \;10^{3}$	3.33 $\times \;10^{3}$	2.96 $\times \;10^{3}$	3.57 $\times \;10^{3}$	2.55 $\times \;10^{3}$	2.73 $\times \;10^{3}$
	mean	3.19 $\times \;10^{3}$	3.75 $\times \;10^{3}$	4.27 $\times \;10^{3}$	4.25 $\times \;10^{3}$	3.20 $\times \;10^{3}$	4.05 $\times \;10^{3}$	**2.98** $\;\times \;10^{3}$	3.14 $\times \;10^{3}$
	std	2.80 $\times \;10^{4}$	1.67 $\times \;10^{4}$	6.47 $\times \;10^{3}$	1.82 $\times \;10^{5}$	1.35 $\times \;10^{4}$	4.95 $\times \;10^{4}$	4.38 $\times \;10^{4}$	3.08 $\times \;10^{4}$
	degree	3	5	8	7	4	6	1	2
F7	min	1.64 $\times \;10^{3}$	2.00 $\times \;10^{3}$	2.62 $\times \;10^{3}$	1.81 $\times \;10^{3}$	1.77 $\times \;10^{3}$	2.29 $\times \;10^{3}$	1.75 $\times \;10^{3}$	1.69 $\times \;10^{3}$
	mean	1.84 $\times \;10^{3}$	2.13 $\times \;10^{3}$	2.77 $\times \;10^{3}$	2.02 $\times \;10^{3}$	1.89 $\times \;10^{3}$	2.43 $\times \;10^{3}$	2.10 $\times \;10^{3}$	**1.79** $\;\times \;10^{3}$
	std	2.58 $\times \;10^{3}$	3.14 $\times \;10^{3}$	7.42 $\times \;10^{3}$	1.65 $\times \;10^{4}$	5.02 $\times \;10^{3}$	4.60 $\times \;10^{3}$	6.04 $\times \;10^{4}$	1.47 $\times \;10^{4}$
	degree	2	6	8	4	3	7	5	1
F8	min	2.42 $\times \;10^{4}$	6.06 $\times \;10^{4}$	8.96 $\times \;10^{4}$	6.11 $\times \;10^{4}$	2.59 $\times \;10^{4}$	6.99 $\times \;10^{4}$	4.75 $\times \;10^{4}$	2.88 $\times \;10^{4}$
	mean	**2.53** $\;\times \;10^{4}$	6.95 $\times \;10^{4}$	1.08 $\times \;10^{5}$	7.36 $\times \;10^{4}$	3.04 $\times \;10^{4}$	8.98 $\times \;10^{4}$	7.60 $\times \;10^{4}$	3.13 $\times \;10^{4}$
	std	3.99 $\times \;10^{5}$	2.19 $\times \;10^{7}$	7.46 $\times \;10^{7}$	7.56 $\times \;10^{7}$	8.36 $\times \;10^{6}$	9.74 $\times \;10^{7}$	1.03 $\times \;10^{8}$	1.45 $\times \;10^{8}$
	degree	1	4	8	5	2	7	6	3
F9	min	1.40 $\times \;10^{4}$	2.05 $\times \;10^{4}$	3.33 $\times \;10^{4}$	2.82 $\times \;10^{4}$	1.75 $\times \;10^{4}$	3.09 $\times \;10^{4}$	1.94 $\times \;10^{4}$	1.70 $\times \;10^{4}$
	mean	**1.73** $\;\times \;10^{4}$	2.47 $\times \;10^{4}$	3.51 $\times \;10^{4}$	3.21 $\times \;10^{4}$	1.94 $\times \;10^{4}$	3.31 $\times \;10^{4}$	2.84 $\times \;10^{4}$	1.79 $\times \;10^{4}$
	std	1.25 $\times \;10^{6}$	5.18 $\times \;10^{6}$	9.98 $\times \;10^{5}$	8.34 $\times \;10^{5}$	1.52 $\times \;10^{6}$	4.17 $\times \;10^{5}$	2.36 $\times \;10^{7}$	1.58 $\times \;10^{7}$
	degree	1	4	8	6	3	7	5	2
F10	min	3.08 $\times \;10^{4}$	7.34 $\times \;10^{4}$	6.10 $\times \;10^{5}$	6.63 $\times \;10^{4}$	1.26 $\times \;10^{4}$	1.34 $\times \;10^{5}$	1.40 $\times \;10^{5}$	4.26 $\times \;10^{4}$
	mean	7.62 $\times \;10^{4}$	1.41 $\times \;10^{5}$	9.69 $\times \;10^{6}$	1.03 $\times \;10^{5}$	**3.57** $\;\times \;10^{4}$	1.85 $\times \;10^{5}$	2.30 $\times \;10^{5}$	5.47 $\times \;10^{4}$
	std	5.12 $\times \;10^{8}$	1.10 $\times \;10^{9}$	1.40 $\times \;10^{15}$	2.89 $\times \;10^{8}$	9.99 $\times \;10^{7}$	9.62 $\times \;10^{8}$	3.30 $\times \;10^{9}$	8.87 $\times \;10^{8}$
	degree	3	5	8	4	1	6	7	2
F11	min	7.17 $\times \;10^{7}$	5.31 $\times \;10^{9}$	2.11 $\times \;10^{11}$	1.01 $\times \;10^{10}$	1.19 $\times \;10^{9}$	7.93 $\times \;10^{10}$	2.95 $\times \;10^{9}$	8.48 $\times \;10^{7}$
	mean	**1.88** $\;\times \;10^{8}$	1.14 $\times \;10^{10}$	2.51 $\times \;10^{11}$	2.05 $\times \;10^{10}$	8.44 $\times \;10^{9}$	1.03 $\times \;10^{11}$	7.34 $\times \;10^{9}$	2.27 $\times \;10^{9}$
	std	4.75 $\times \;10^{15}$	2.29 $\times \;10^{19}$	2.12 $\times \;10^{20}$	3.51 $\times \;10^{19}$	4.61 $\times \;10^{19}$	1.32 $\times \;10^{20}$	4.52 $\times \;10^{18}$	1.02 $\times \;10^{19}$
	degree	1	5	8	6	4	7	3	2
F12	min	2.24 $\times \;10^{4}$	6.36 $\times \;10^{7}$	5.08 $\times \;10^{10}$	3.47 $\times \;10^{8}$	4.79 $\times \;10^{5}$	9.56 $\times \;10^{9}$	1.81 $\times \;10^{7}$	7.67 $\times \;10^{4}$
	mean	**6.46** $\;\times \;10^{4}$	2.32 $\times \;10^{8}$	6.25 $\times \;10^{10}$	1.21 $\times \;10^{9}$	1.52 $\times \;10^{8}$	1.69 $\times \;10^{10}$	3.22 $\times \;10^{8}$	8.89 $\times \;10^{5}$
	std	9.55 $\times \;10^{9}$	3.62 $\times \;10^{16}$	1.52 $\times \;10^{19}$	9.75 $\times \;10^{17}$	7.87 $\times \;10^{16}$	1.83 $\times \;10^{19}$	4.87 $\times \;10^{16}$	3.46 $\times \;10^{13}$
	degree	1	4	8	6	3	7	5	2
F13	min	8.28 $\times \;10^{5}$	4.89 $\times \;10^{6}$	1.09 $\times \;10^{8}$	1.18 $\times \;10^{6}$	5.08 $\times \;10^{5}$	1.76 $\times \;10^{7}$	2.85 $\times \;10^{6}$	1.01 $\times \;10^{6}$
	mean	2.10 $\times \;10^{6}$	1.12 $\times \;10^{7}$	4.40 $\times \;10^{8}$	3.96 $\times \;10^{6}$	**1.72** $\;\times \;10^{6}$	6.25 $\times \;10^{7}$	2.00 $\times \;10^{7}$	2.05 $\times \;10^{6}$
	std	7.27 $\times \;10^{11}$	1.19 $\times \;10^{13}$	5.79 $\times \;10^{16}$	4.22 $\times \;10^{12}$	8.34 $\times \;10^{11}$	8.33 $\times \;10^{14}$	1.68 $\times \;10^{14}$	1.39 $\times \;10^{13}$
	degree	3	5	8	4	1	7	6	2
F14	min	9.59 $\times \;10^{3}$	6.90 $\times \;10^{6}$	1.99 $\times \;10^{10}$	6.79 $\times \;10^{6}$	1.22 $\times \;10^{4}$	3.10 $\times \;10^{9}$	1.81 $\times \;10^{5}$	2.64 $\times \;10^{4}$
	mean	**2.21** $\;\times \;10^{4}$	2.12 $\times \;10^{7}$	3.58 $\times \;10^{10}$	4.95 $\times \;10^{7}$	6.13 $\times \;10^{6}$	5.92 $\times \;10^{9}$	1.02 $\times \;10^{8}$	3.89 $\times \;10^{5}$
	std	1.43 $\times \;10^{8}$	6.12 $\times \;10^{14}$	3.02 $\times \;10^{19}$	2.07 $\times \;10^{15}$	4.58 $\times \;10^{14}$	2.94 $\times \;10^{18}$	2.49 $\times \;10^{16}$	1.05 $\times \;10^{12}$
	degree	1	4	8	5	3	7	6	2
F15	min	4.61 $\times \;10^{3}$	7.96 $\times \;10^{3}$	1.61 $\times \;10^{4}$	7.59 $\times \;10^{3}$	5.48 $\times \;10^{3}$	1.30 $\times \;10^{4}$	7.13 $\times \;10^{3}$	4.05 $\times \;10^{3}$
	mean	6.13 $\times \;10^{3}$	1.02 $\times \;10^{4}$	2.42 $\times \;10^{4}$	9.95 $\times \;10^{3}$	7.39 $\times \;10^{3}$	1.49 $\times \;10^{4}$	9.28 $\times \;10^{3}$	**5.77** $\;\times \;10^{3}$
	std	6.18 $\times \;10^{5}$	1.43 $\times \;10^{6}$	9.69 $\times \;10^{6}$	1.58 $\times \;10^{6}$	1.08 $\times \;10^{6}$	6.52 $\times \;10^{5}$	1.82 $\times \;10^{6}$	1.14 $\times \;10^{6}$
	degree	2	6	8	5	3	7	4	1
F16	min	4.82 $\times \;10^{3}$	6.14 $\times \;10^{3}$	3.10 $\times \;10^{6}$	4.64 $\times \;10^{3}$	5.02 $\times \;10^{3}$	1.41 $\times \;10^{4}$	7.59 $\times \;10^{3}$	3.02 $\times \;10^{3}$
	mean	6.03 $\times \;10^{3}$	9.15 $\times \;10^{3}$	3.46 $\times \;10^{7}$	6.62 $\times \;10^{3}$	6.82 $\times \;10^{3}$	9.05 $\times \;10^{4}$	9.57 $\times \;10^{3}$	**5.71** $\;\times \;10^{3}$
	std	4.70 $\times \;10^{5}$	6.13 $\times \;10^{7}$	1.25 $\times \;10^{15}$	7.55 $\times \;10^{5}$	1.18 $\times \;10^{6}$	9.12 $\times \;10^{9}$	4.04 $\times \;10^{6}$	1.10 $\times \;10^{6}$
	degree	2	5	8	3	4	7	6	1
F17	min	3.81 $\times \;10^{5}$	2.54 $\times \;10^{6}$	1.92 $\times \;10^{8}$	1.05 $\times \;10^{6}$	1.05 $\times \;10^{6}$	4.79 $\times \;10^{7}$	3.84 $\times \;10^{6}$	1.58 $\times \;10^{6}$
	mean	2.67 $\times \;10^{6}$	9.22 $\times \;10^{6}$	7.50 $\times \;10^{8}$	6.03 $\times \;10^{6}$	2.93 $\times \;10^{6}$	1.34 $\times \;10^{8}$	1.98 $\times \;10^{7}$	**1.78** $\;\times \;10^{6}$
	std	1.41 $\times \;10^{12}$	1.84 $\times \;10^{13}$	1.27 $\times \;10^{17}$	1.22 $\times \;10^{13}$	3.40 $\times \;10^{12}$	2.91 $\times \;10^{15}$	1.11 $\times \;10^{14}$	1.23 $\times \;10^{13}$
	degree	2	5	8	4	3	7	6	1
F18	min	2.88 $\times \;10^{3}$	1.33 $\times \;10^{7}$	2.44 $\times \;10^{10}$	4.57 $\times \;10^{6}$	7.51 $\times \;10^{4}$	3.14 $\times \;10^{9}$	1.43 $\times \;10^{7}$	4.15 $\times \;10^{4}$
	mean	2.88 $\times \;10^{4}$	3.92 $\times \;10^{7}$	3.44 $\times \;10^{10}$	4.83 $\times \;10^{7}$	1.27 $\times \;10^{7}$	5.29 $\times \;10^{9}$	1.48 $\times \;10^{8}$	**1.47** $\;\times \;10^{6}$
	std	5.28 $\times \;10^{9}$	3.32 $\times \;10^{14}$	2.75 $\times \;10^{19}$	1.31 $\times \;10^{15}$	2.93 $\times \;10^{15}$	1.82 $\times \;10^{18}$	1.43 $\times \;10^{16}$	1.33 $\times \;10^{12}$
	degree	1	4	8	5	3	7	6	2
F19	min	4.58 $\times \;10^{3}$	4.92 $\times \;10^{3}$	8.72 $\times \;10^{3}$	6.65 $\times \;10^{3}$	4.16 $\times \;10^{3}$	7.41 $\times \;10^{3}$	5.45 $\times \;10^{3}$	4.02 $\times \;10^{3}$
	mean	6.06 $\times \;10^{3}$	6.12 $\times \;10^{3}$	9.25 $\times \;10^{3}$	7.46 $\times \;10^{3}$	5.25 $\times \;10^{3}$	8.13 $\times \;10^{3}$	7.28 $\times \;10^{3}$	**5.25** $\;\times \;10^{3}$
	std	3.59 $\times \;10^{5}$	2.69 $\times \;10^{5}$	6.94 $\times \;10^{4}$	1.11 $\times \;10^{5}$	3.38 $\times \;10^{5}$	1.12 $\times \;10^{5}$	6.12 $\times \;10^{5}$	5.82 $\times \;10^{5}$
	degree	3	4	8	6	2	7	5	1
F20	min	3.35 $\times \;10^{3}$	4.03 $\times \;10^{3}$	4.46 $\times \;10^{3}$	3.18 $\times \;10^{3}$	3.33 $\times \;10^{3}$	3.99 $\times \;10^{3}$	3.73 $\times \;10^{3}$	3.03 $\times \;10^{3}$
	mean	3.66 $\times \;10^{3}$	4.40 $\times \;10^{3}$	4.95 $\times \;10^{3}$	3.44 $\times \;10^{3}$	3.59 $\times \;10^{3}$	4.22 $\times \;10^{3}$	4.04 $\times \;10^{3}$	**3.39** $\;\times \;10^{3}$
	std	4.70 $\times \;10^{4}$	3.33 $\times \;10^{4}$	4.68 $\times \;10^{4}$	2.01 $\times \;10^{4}$	2.37 $\times \;10^{4}$	1.17 $\times \;10^{4}$	3.72 $\times \;10^{4}$	4.85 $\times \;10^{4}$
	degree	4	7	8	2	3	6	5	1
F21	min	1.40 $\times \;10^{4}$	2.45 $\times \;10^{4}$	3.54 $\times \;10^{4}$	3.31 $\times \;10^{4}$	1.85 $\times \;10^{4}$	3.38 $\times \;10^{4}$	2.07 $\times \;10^{4}$	2.01 $\times \;10^{4}$
	mean	**1.93** $\;\times \;10^{4}$	2.77 $\times \;10^{4}$	3.75 $\times \;10^{4}$	3.48 $\times \;10^{4}$	2.34 $\times \;10^{4}$	3.53 $\times \;10^{4}$	2.82 $\times \;10^{4}$	2.72 $\times \;10^{4}$
	std	2.89 $\times \;10^{6}$	2.00 $\times \;10^{6}$	1.30 $\times \;10^{6}$	4.73 $\times \;10^{5}$	2.95 $\times \;10^{6}$	6.18 $\times \;10^{5}$	2.46 $\times \;10^{7}$	9.05 $\times \;10^{6}$
	degree	1	4	8	6	2	7	5	3
F22	min	3.75 $\times \;10^{3}$	5.43 $\times \;10^{3}$	5.55 $\times \;10^{3}$	3.93 $\times \;10^{3}$	3.98 $\times \;10^{3}$	5.05 $\times \;10^{3}$	4.49 $\times \;10^{3}$	3.75 $\times \;10^{3}$
	mean	4.25 $\times \;10^{3}$	5.94 $\times \;10^{3}$	6.53 $\times \;10^{3}$	4.20 $\times \;10^{3}$	4.58 $\times \;10^{3}$	5.26 $\times \;10^{3}$	4.81 $\times \;10^{3}$	**4.17** $\;\times \;10^{3}$
	std	3.78 $\times \;10^{4}$	1.34 $\times \;10^{5}$	4.28 $\times \;10^{5}$	2.43 $\times \;10^{4}$	8.34 $\times \;10^{4}$	1.69 $\times \;10^{4}$	3.07 $\times \;10^{4}$	1.14 $\times \;10^{5}$
	degree	3	7	8	2	4	6	5	1
F23	min	4.56 $\times \;10^{3}$	7.05 $\times \;10^{3}$	7.35 $\times \;10^{3}$	5.23 $\times \;10^{3}$	5.43 $\times \;10^{3}$	6.93 $\times \;10^{3}$	5.28 $\times \;10^{3}$	4.91 $\times \;10^{3}$
	mean	5.22 $\times \;10^{3}$	8.58 $\times \;10^{3}$	9.44 $\times \;10^{3}$	5.91 $\times \;10^{3}$	5.97 $\times \;10^{3}$	7.44 $\times \;10^{3}$	6.30 $\times \;10^{3}$	5.19 $\times \;10^{3}$
	std	1.26 $\times \;10^{5}$	2.60 $\times \;10^{5}$	3.71 $\times \;10^{6}$	1.86 $\times \;10^{5}$	9.61 $\times \;10^{4}$	8.67 $\times \;10^{4}$	2.41 $\times \;10^{5}$	**2.55** $\;\times \;10^{5}$
	degree	2	7	8	3	4	6	5	1
F24	min	3.54 $\times \;10^{3}$	5.91 $\times \;10^{3}$	2.47 $\times \;10^{4}$	9.14 $\times \;10^{3}$	5.46 $\times \;10^{3}$	1.82 $\times \;10^{4}$	5.20 $\times \;10^{3}$	4.73 $\times \;10^{3}$
	mean	**3.68** $\;\times \;10^{3}$	6.78 $\times \;10^{3}$	3.22 $\times \;10^{4}$	1.23 $\times \;10^{4}$	7.66 $\times \;10^{3}$	2.29 $\times \;10^{4}$	1.15 $\times \;10^{4}$	5.71 $\times \;10^{3}$
	std	6.90 $\times \;10^{3}$	2.16 $\times \;10^{5}$	9.35 $\times \;10^{6}$	5.86 $\times \;10^{6}$	1.11 $\times \;10^{6}$	5.41 $\times \;10^{6}$	5.86 $\times \;10^{7}$	7.74 $\times \;10^{5}$
	degree	1	3	8	6	4	7	5	2
F25	min	4.86 $\times \;10^{3}$	2.83 $\times \;10^{4}$	5.06 $\times \;10^{4}$	2.95 $\times \;10^{4}$	2.64 $\times \;10^{4}$	3.46 $\times \;10^{4}$	1.97 $\times \;10^{4}$	2.01 $\times \;10^{4}$
	mean	**2.09** $\;\times \;10^{4}$	3.22 $\times \;10^{4}$	5.81 $\times \;10^{4}$	3.64 $\times \;10^{4}$	3.17 $\times \;10^{4}$	4.15 $\times \;10^{4}$	2.62 $\times \;10^{4}$	2.61 $\times \;10^{4}$
	std	5.17 $\times \;10^{7}$	6.14 $\times \;10^{6}$	1.33 $\times \;10^{7}$	1.43 $\times \;10^{7}$	6.11 $\times \;10^{6}$	8.65 $\times \;10^{6}$	1.29 $\times \;10^{7}$	1.64 $\times \;10^{7}$
	degree	1	5	8	6	4	7	3	2
F26	min	3.62 $\times \;10^{3}$	5.85 $\times \;10^{3}$	8.14 $\times \;10^{3}$	4.71 $\times \;10^{3}$	4.34 $\times \;10^{3}$	7.44 $\times \;10^{3}$	4.02 $\times \;10^{3}$	3.70 $\times \;10^{3}$
	mean	**3.89** $\;\times \;10^{3}$	7.65 $\times \;10^{3}$	1.16 $\times \;10^{4}$	5.47 $\times \;10^{3}$	5.41 $\times \;10^{3}$	8.71 $\times \;10^{3}$	4.63 $\times \;10^{3}$	4.59 $\times \;10^{3}$
	std	6.24 $\times \;10^{4}$	2.58 $\times \;10^{6}$	2.17 $\times \;10^{6}$	2.17 $\times \;10^{5}$	3.43 $\times \;10^{5}$	4.26 $\times \;10^{5}$	2.51 $\times \;10^{5}$	2.79 $\times \;10^{5}$
	degree	1	6	8	5	4	7	3	2
F27	min	3.65 $\times \;10^{3}$	7.61 $\times \;10^{3}$	3.03 $\times \;10^{4}$	1.09 $\times \;10^{4}$	6.81 $\times \;10^{3}$	2.19 $\times \;10^{4}$	7.03 $\times \;10^{3}$	4.87 $\times \;10^{3}$
	mean	**3.80** $\;\times \;10^{3}$	9.26 $\times \;10^{3}$	3.71 $\times \;10^{4}$	1.47 $\times \;10^{4}$	9.62 $\times \;10^{3}$	2.67 $\times \;10^{4}$	1.93 $\times \;10^{4}$	7.56 $\times \;10^{3}$
	std	9.55 $\times \;10^{3}$	7.57 $\times \;10^{5}$	6.98 $\times \;10^{6}$	3.79 $\times \;10^{6}$	2.39 $\times \;10^{6}$	4.12 $\times \;10^{6}$	3.81 $\times \;10^{7}$	2.37 $\times \;10^{6}$
	degree	1	3	8	5	4	7	6	2
F28	min	6.75 $\times \;10^{3}$	1.04 $\times \;10^{4}$	2.25 $\times \;10^{5}$	9.23 $\times \;10^{3}$	8.58 $\times \;10^{3}$	2.14 $\times \;10^{4}$	9.10 $\times \;10^{3}$	5.66 $\times \;10^{3}$
	mean	**7.74** $\;\times \;10^{3}$	1.31 $\times \;10^{4}$	1.41 $\times \;10^{6}$	1.27 $\times \;10^{4}$	1.05 $\times \;10^{4}$	4.16 $\times \;10^{4}$	1.23 $\times \;10^{4}$	9.82 $\times \;10^{3}$
	std	2.40 $\times \;10^{5}$	2.52 $\times \;10^{6}$	1.07 $\times \;10^{12}$	3.17 $\times \;10^{6}$	7.99 $\times \;10^{5}$	6.39 $\times \;10^{8}$	2.56 $\times \;10^{6}$	8.12 $\times \;10^{5}$
	degree	1	6	8	5	3	7	4	2
F29	min	1.89 $\times \;10^{5}$	3.34 $\times \;10^{8}$	4.47 $\times \;10^{10}$	3.43 $\times \;10^{8}$	8.25 $\times \;10^{6}$	6.19 $\times \;10^{9}$	6.66 $\times \;10^{7}$	5.10 $\times \;10^{6}$
	mean	**6.17** $\;\times \;10^{5}$	7.94 $\times \;10^{8}$	5.67 $\times \;10^{10}$	2.10 $\times \;10^{9}$	1.36 $\times \;10^{8}$	1.33 $\times \;10^{10}$	2.66 $\times \;10^{8}$	7.45 $\times \;10^{7}$
	std	1.28 $\times \;10^{11}$	1.03 $\times \;10^{17}$	2.23 $\times \;10^{19}$	2.76 $\times \;10^{18}$	3.09 $\times \;10^{16}$	9.89 $\times \;10^{18}$	1.95 $\times \;10^{16}$	6.57 $\times \;10^{16}$
	degree	1	5	8	6	3	7	4	2

**Table 5:** Wilcoxon rank-sum test (Dim = 30).

	SSA	HHO	BOA	OMA	WOA	SCA	DBO
F1	3.02 $\times \;10^{-11}$	2.19 $\times \;10^{-8}$	2.77 $\times \;10^{-11}$	7.18 $\times \;10^{-11}$	1.34 $\times \;10^{-5}$	3.02 $\times \;10^{-11}$	3.02 $\times \;10^{-7}$
F2	3.99 $\times \;10^{-4}$	8.42 $\times \;10^{-1}$	3.34 $\times \;10^{-11}$	1.15 $\times \;10^{-1}$	3.02 $\times \;10^{-11}$	4.18 $\times \;10^{-9}$	3.02 $\times \;10^{-9}$
F3	8.20 $\times \;10^{-7}$	3.16 $\times \;10^{-10}$	3.02 $\times \;10^{-11}$	4.62 $\times \;10^{-10}$	5.01 $\times \;10^{-2}$	3.02 $\times \;10^{-11}$	3.02 $\times \;10^{-6}$
F4	4.03 $\times \;10^{-3}$	5.19 $\times \;10^{-7}$	3.02 $\times \;10^{-11}$	7.96 $\times \;10^{-1}$	6.79 $\times \;10^{-2}$	8.15 $\times \;10^{-11}$	3.02 $\times \;10^{-5}$
F5	1.30 $\times \;10^{-1}$	2.37 $\times \;10^{-10}$	3.02 $\times \;10^{-11}$	9.53 $\times \;10^{-7}$	3.33 $\times \;10^{-1}$	1.17 $\times \;10^{-9}$	1.87 $\times \;10^{-3}$
F6	1.11 $\times \;10^{-6}$	8.89 $\times \;10^{-10}$	3.02 $\times \;10^{-11}$	1.06 $\times \;10^{-3}$	7.24 $\times \;10^{-2}$	2.20 $\times \;10^{-7}$	3.51 $\times \;10^{-2}$
F7	3.64 $\times \;10^{-2}$	7.30 $\times \;10^{-4}$	3.02 $\times \;10^{-11}$	4.68 $\times \;10^{-2}$	6.20 $\times \;10^{-4}$	4.08 $\times \;10^{-11}$	2.28 $\times \;10^{-5}$
F8	5.20 $\times \;10^{-1}$	4.20 $\times \;10^{-10}$	3.02 $\times \;10^{-11}$	2.61 $\times \;10^{-2}$	1.22 $\times \;10^{-2}$	6.28 $\times \;10^{-6}$	1.44 $\times \;10^{-2}$
F9	6.28 $\times \;10^{-6}$	4.21 $\times \;10^{-2}$	3.02 $\times \;10^{-11}$	3.50 $\times \;10^{-9}$	1.16 $\times \;10^{-7}$	2.87 $\times \;10^{-10}$	7.35 $\times \;10^{-1}$
F10	2.16 $\times \;10^{-3}$	6.75 $\times \;10^{-10}$	3.02 $\times \;10^{-11}$	7.53 $\times \;10^{-1}$	4.03 $\times \;10^{-3}$	3.02 $\times \;10^{-11}$	1.31 $\times \;10^{-8}$
F11	2.98 $\times \;10^{-8}$	1.69 $\times \;10^{-9}$	3.02 $\times \;10^{-11}$	2.46 $\times \;10^{-2}$	1.04 $\times \;10^{-4}$	3.02 $\times \;10^{-11}$	8.56 $\times \;10^{-4}$
F12	5.75 $\times \;10^{-2}$	1.43 $\times \;10^{-8}$	3.02 $\times \;10^{-11}$	6.20 $\times \;10^{-1}$	2.25 $\times \;10^{-4}$	3.02 $\times \;10^{-11}$	8.48 $\times \;10^{-9}$
F13	1.49 $\times \;10^{-4}$	8.15 $\times \;10^{-5}$	3.02 $\times \;10^{-11}$	8.20 $\times \;10^{-7}$	8.15 $\times \;10^{-11}$	6.53 $\times \;10^{-8}$	6.00 $\times \;10^{-1}$
F14	6.35 $\times \;10^{-2}$	8.15 $\times \;10^{-11}$	3.02 $\times \;10^{-11}$	2.17 $\times \;10^{-1}$	6.20 $\times \;10^{-4}$	3.02 $\times \;10^{-11}$	6.01 $\times \;10^{-8}$
F15	5.55 $\times \;10^{-2}$	4.57 $\times \;10^{-9}$	3.02 $\times \;10^{-11}$	3.09 $\times \;10^{-6}$	1.27 $\times \;10^{-2}$	4.50 $\times \;10^{-11}$	3.59 $\times \;10^{-5}$
F16	6.41 $\times \;10^{-1}$	1.17 $\times \;10^{-2}$	3.69 $\times \;10^{-11}$	5.97 $\times \;10^{-5}$	1.44 $\times \;10^{-3}$	1.06 $\times \;10^{-3}$	3.39 $\times \;10^{-2}$
F17	4.64 $\times \;10^{-1}$	1.29 $\times \;10^{-6}$	3.02 $\times \;10^{-11}$	1.27 $\times \;10^{-2}$	1.73 $\times \;10^{-7}$	3.34 $\times \;10^{-11}$	1.44 $\times \;10^{-3}$
F18	3.27 $\times \;10^{-2}$	3.02 $\times \;10^{-11}$	3.02 $\times \;10^{-11}$	7.45 $\times \;10^{-1}$	1.44 $\times \;10^{-2}$	3.02 $\times \;10^{-11}$	4.31 $\times \;10^{-8}$
F19	5.27 $\times \;10^{-5}$	6.77 $\times \;10^{-5}$	3.34 $\times \;10^{-11}$	8.56 $\times \;10^{-2}$	1.33 $\times \;10^{-1}$	1.31 $\times \;10^{-8}$	3.02 $\times \;10^{-3}$
F20	9.47 $\times \;10^{-1}$	7.77 $\times \;10^{-9}$	3.02 $\times \;10^{-11}$	3.78 $\times \;10^{-2}$	6.97 $\times \;10^{-3}$	1.29 $\times \;10^{-9}$	5.27 $\times \;10^{-5}$
F21	2.07 $\times \;10^{-2}$	3.82 $\times \;10^{-9}$	4.98 $\times \;10^{-11}$	1.87 $\times \;10^{-5}$	1.50 $\times \;10^{-2}$	1.78 $\times \;10^{-10}$	3.01 $\times \;10^{-4}$
F22	1.76 $\times \;10^{-2}$	7.39 $\times \;10^{-11}$	3.69 $\times \;10^{-11}$	4.98 $\times \;10^{-4}$	4.73 $\times \;10^{-1}$	1.60 $\times \;10^{-7}$	9.33 $\times \;10^{-2}$
F23	5.19 $\times \;10^{-2}$	1.17 $\times \;10^{-9}$	1.46 $\times \;10^{-10}$	1.91 $\times \;10^{-1}$	7.55 $\times \;10^{-1}$	6.53 $\times \;10^{-7}$	1.91 $\times \;10^{-1}$
F24	8.85 $\times \;10^{-11}$	4.11 $\times \;10^{-7}$	3.02 $\times \;10^{-11}$	6.07 $\times \;10^{-11}$	7.56 $\times \;10^{-1}$	3.02 $\times \;10^{-11}$	7.98 $\times \;10^{-2}$
F25	5.33 $\times \;10^{-2}$	2.19 $\times \;10^{-8}$	4.98 $\times \;10^{-11}$	4.92 $\times \;10^{-1}$	5.86 $\times \;10^{-1}$	7.04 $\times \;10^{-7}$	4.64 $\times \;10^{-3}$
F26	1.76 $\times \;10^{-4}$	2.92 $\times \;10^{-9}$	3.02 $\times \;10^{-11}$	9.12 $\times \;10^{-1}$	3.95 $\times \;10^{-1}$	1.55 $\times \;10^{-9}$	9.47 $\times \;10^{-1}$
F27	1.61 $\times \;10^{-10}$	1.61 $\times \;10^{-6}$	3.02 $\times \;10^{-11}$	2.03 $\times \;10^{-9}$	4.12 $\times \;10^{-1}$	3.02 $\times \;10^{-11}$	2.13 $\times \;10^{-5}$
F28	8.31 $\times \;10^{-3}$	1.25 $\times \;10^{-5}$	3.02 $\times \;10^{-11}$	9.33 $\times \;10^{-2}$	6.41 $\times \;10^{-1}$	1.70 $\times \;10^{-8}$	8.24 $\times \;10^{-2}$
F29	2.88 $\times \;10^{-10}$	3.02 $\times \;10^{-10}$	3.02 $\times \;10^{-11}$	7.06 $\times \;10^{-1}$	2.15 $\times \;10^{-10}$	3.02 $\times \;10^{-11}$	3.83 $\times \;10^{-6}$

**Table 6:** Wilcoxon rank-sum test (Dim = 100).

	SSA	HHO	BOA	OMA	WOA	SCA	DBO
F1	9.47 $\times \;10^{-1}$	3.02 $\times \;10^{-11}$	3.02 $\times \;10^{-11}$	3.02 $\times \;10^{-11}$	9.12 $\times \;10^{-1}$	3.02 $\times \;10^{-11}$	1.21 $\times \;10^{-10}$
F2	1.87 $\times \;10^{-5}$	2.24 $\times \;10^{-2}$	3.02 $\times \;10^{-11}$	4.94 $\times \;10^{-5}$	3.02 $\times \;10^{-11}$	5.00 $\times \;10^{-9}$	3.34 $\times \;10^{-11}$
F3	4.73 $\times \;10^{-1}$	2.03 $\times \;10^{-7}$	3.02 $\times \;10^{-11}$	2.03 $\times \;10^{-9}$	4.73 $\times \;10^{-1}$	3.02 $\times \;10^{-11}$	1.87 $\times \;10^{-5}$
F4	1.29 $\times \;10^{-6}$	6.01 $\times \;10^{-8}$	3.02 $\times \;10^{-11}$	2.17 $\times \;10^{-1}$	1.00 $\times \;10^{0}$	1.21 $\times \;10^{-10}$	2.51 $\times \;10^{-2}$
F5	3.02 $\times \;10^{-10}$	3.34 $\times \;10^{-11}$	3.02 $\times \;10^{-11}$	3.02 $\times \;10^{-10}$	2.68 $\times \;10^{-6}$	4.50 $\times \;10^{-11}$	6.20 $\times \;10^{-1}$
F6	3.02 $\times \;10^{-11}$	3.02 $\times \;10^{-11}$	3.02 $\times \;10^{-11}$	3.01 $\times \;10^{-7}$	1.32 $\times \;10^{-4}$	3.02 $\times \;10^{-11}$	2.71 $\times \;10^{-1}$
F7	6.91 $\times \;10^{-4}$	1.22 $\times \;10^{-2}$	3.02 $\times \;10^{-11}$	6.10 $\times \;10^{-1}$	1.12 $\times \;10^{-2}$	8.99 $\times \;10^{-11}$	2.84 $\times \;10^{-4}$
F8	4.98 $\times \;10^{-11}$	4.08 $\times \;10^{-11}$	3.02 $\times \;10^{-11}$	1.39 $\times \;10^{-6}$	9.12 $\times \;10^{-1}$	6.07 $\times \;10^{-11}$	2.15 $\times \;10^{-6}$
F9	8.19 $\times \;10^{-1}$	1.17 $\times \;10^{-2}$	3.02 $\times \;10^{-11}$	4.08 $\times \;10^{-11}$	6.91 $\times \;10^{-4}$	3.02 $\times \;10^{-11}$	9.82 $\times \;10^{-1}$
F10	1.44 $\times \;10^{-3}$	3.26 $\times \;10^{-1}$	3.02 $\times \;10^{-11}$	3.67 $\times \;10^{-3}$	3.83 $\times \;10^{-5}$	3.02 $\times \;10^{-11}$	6.53 $\times \;10^{-8}$
F11	8.66 $\times \;10^{-3}$	1.21 $\times \;10^{-10}$	3.02 $\times \;10^{-11}$	2.53 $\times \;10^{-4}$	3.37 $\times \;10^{-5}$	3.02 $\times \;10^{-11}$	5.97 $\times \;10^{-9}$
F12	4.85 $\times \;10^{-5}$	1.36 $\times \;10^{-7}$	3.02 $\times \;10^{-11}$	9.47 $\times \;10^{-3}$	2.49 $\times \;10^{-6}$	3.02 $\times \;10^{-11}$	1.43 $\times \;10^{-5}$
F13	8.13 $\times \;10^{-2}$	5.09 $\times \;10^{-8}$	3.02 $\times \;10^{-11}$	3.34 $\times \;10^{-3}$	3.02 $\times \;10^{-11}$	8.99 $\times \;10^{-11}$	1.54 $\times \;10^{-1}$
F14	5.08 $\times \;10^{-3}$	2.20 $\times \;10^{-7}$	3.02 $\times \;10^{-11}$	1.05 $\times \;10^{-1}$	1.17 $\times \;10^{-5}$	3.02 $\times \;10^{-11}$	1.68 $\times \;10^{-4}$
F15	5.49 $\times \;10^{-1}$	3.77 $\times \;10^{-4}$	3.02 $\times \;10^{-11}$	1.12 $\times \;10^{-2}$	3.02 $\times \;10^{-5}$	1.09 $\times \;10^{-10}$	3.26 $\times \;10^{-1}$
F16	3.13 $\times \;10^{-4}$	9.79 $\times \;10^{-5}$	3.02 $\times \;10^{-11}$	2.16 $\times \;10^{-3}$	2.42 $\times \;10^{-2}$	1.55 $\times \;10^{-6}$	7.88 $\times \;10^{-3}$
F17	4.36 $\times \;10^{-2}$	8.66 $\times \;10^{-5}$	3.02 $\times \;10^{-11}$	9.23 $\times \;10^{-1}$	2.03 $\times \;10^{-9}$	3.34 $\times \;10^{-11}$	6.90 $\times \;10^{-3}$
F18	2.58 $\times \;10^{-1}$	2.44 $\times \;10^{-9}$	3.02 $\times \;10^{-11}$	3.02 $\times \;10^{-2}$	1.99 $\times \;10^{-2}$	3.02 $\times \;10^{-11}$	3.02 $\times \;10^{-3}$
F19	2.71 $\times \;10^{-1}$	2.51 $\times \;10^{-2}$	3.02 $\times \;10^{-11}$	4.68 $\times \;10^{-2}$	2.28 $\times \;10^{-5}$	4.71 $\times \;10^{-4}$	9.93 $\times \;10^{-2}$
F20	5.11 $\times \;10^{-1}$	2.13 $\times \;10^{-5}$	3.02 $\times \;10^{-11}$	7.60 $\times \;10^{-7}$	8.88 $\times \;10^{-6}$	1.46 $\times \;10^{-10}$	8.42 $\times \;10^{-1}$
F21	3.67 $\times \;10^{-3}$	8.12 $\times \;10^{-4}$	4.50 $\times \;10^{-11}$	6.79 $\times \;10^{-2}$	1.37 $\times \;10^{-3}$	1.73 $\times \;10^{-7}$	4.23 $\times \;10^{-3}$
F22	1.81 $\times \;10^{-1}$	8.15 $\times \;10^{-11}$	3.02 $\times \;10^{-11}$	9.21 $\times \;10^{-5}$	2.12 $\times \;10^{-1}$	4.62 $\times \;10^{-10}$	5.30 $\times \;10^{-1}$
F23	1.55 $\times \;10^{-4}$	3.34 $\times \;10^{-11}$	3.02 $\times \;10^{-11}$	3.48 $\times \;10^{-1}$	1.67 $\times \;10^{-1}$	5.49 $\times \;10^{-11}$	5.55 $\times \;10^{-2}$
F24	3.63 $\times \;10^{-1}$	1.25 $\times \;10^{-7}$	3.02 $\times \;10^{-11}$	3.34 $\times \;10^{-11}$	2.42 $\times \;10^{-2}$	3.02 $\times \;10^{-11}$	1.47 $\times \;10^{-7}$
F25	1.54 $\times \;10^{-1}$	3.59 $\times \;10^{-5}$	3.02 $\times \;10^{-11}$	1.06 $\times \;10^{-3}$	3.33 $\times \;10^{-1}$	5.60 $\times \;10^{-7}$	1.22 $\times \;10^{-1}$
F26	3.18 $\times \;10^{-1}$	1.78 $\times \;10^{-10}$	3.02 $\times \;10^{-11}$	2.27 $\times \;10^{-3}$	3.16 $\times \;10^{-5}$	3.02 $\times \;10^{-11}$	1.81 $\times \;10^{-1}$
F27	2.55 $\times \;10^{-1}$	9.76 $\times \;10^{-10}$	3.02 $\times \;10^{-11}$	4.62 $\times \;10^{-10}$	2.58 $\times \;10^{-1}$	3.02 $\times \;10^{-11}$	2.03 $\times \;10^{-9}$
F28	4.86 $\times \;10^{-3}$	2.03 $\times \;10^{-9}$	3.02 $\times \;10^{-11}$	5.49 $\times \;10^{-1}$	2.77 $\times \;10^{-1}$	3.69 $\times \;10^{-11}$	7.77 $\times \;10^{-1}$
F29	2.89 $\times \;10^{-3}$	8.15 $\times \;10^{-11}$	3.02 $\times \;10^{-11}$	1.12 $\times \;10^{-1}$	1.34 $\times \;10^{-5}$	3.02 $\times \;10^{-11}$	5.89 $\times \;10^{-4}$

**Table 7:** Statistical measurement analysis of the tension (compression) spring design.

	SSA	HHO	BOA	OMA	WOA	SCA	DBO	ERDBO
mean	0.012767	0.013597	0.013193	0.012765	0.012666	0.013799	0.013796	0.012665
std	5.1 $\times \;10^{-7}$	1.05 $\times \;10^{-6}$	1.23 $\times \;10^{-8}$	5.84 $\times \;10^{-9}$	7.91 $\times \;10^{-13}$	7.76 $\times \;10^{-6}$	2.55 $\times \;10^{-6}$	1.05 $\times \;10^{-5}$
min	0.012874	0.012666	0.013093	0.012674	0.012665	0.012803	0.012686	0.012665
max	0.017697	0.017206	0.013993	0.012967	0.012669	0.017563	0.017781	0.012666
ranking	4	6	5	3	2	8	7	1

**Table 8:** Statistical measurement analysis of the triangle truss design.

	SSA	HHO	BOA	OMA	WOA	SCA	DBO	ERDBO
mean	263.8988	264.0088	274.8039	263.8960	263.8959	263.8959	263.9041	263.8959
std	7.31 $\times \;10^{-6}$	1.14 $\times \;10^{-2}$	3.03 $\times \;10^{1}$	3.85 $\times \;10^{-8}$	6.69 $\times \;10^{-28}$	8.77 $\times \;10^{-6}$	3.53 $\times \;10^{-4}$	3.78 $\times \;10^{-28}$
min	263.8959	263.8989	265.2901	263.8958	263.8958	263.8960	263.8958	263.8958
max	263.8999	264.2654	282.8427	263.8968	263.8961	263.9023	263.9964	263.8980
ranking	5	7	8	3	2	4	6	1

**Table 9:** Statistical measurement analysis of the triangle truss design.

	SSA	HHO	BOA	OMA	WOA	SCA	DBO	ERDBO
mean	5971.892	6822.387	6960.026	5964.435	5889.333	5997.828	6661.39	5889.301
std	6.60 $\times \;10^{5}$	1.99 $\times \;10^{5}$	2.74 $\times \;10^{11}$	3.32 $\times \;10^{3}$	6.24 $\times \;10^{-11}$	6.65 $\times \;10^{5}$	3.48 $\times \;10^{6}$	1.76 $\times \;10^{-5}$
min	5889.604	6089.84	76,052.22	5887.563	5885.333	5889.330	5885.333	5885.333
max	6401.958	7753.804	2,532,499	6076.547	5899.333	6969.001	16,043.15	7321.479
ranking	4	7	8	3	2	5	6	1

## Data Availability

The data presented in this study are available within the article. Further inquiries can be directed to the corresponding author.
